# Simultaneous Inhibition of EGFR/VEGFR and Cyclooxygenase-2 Targets Stemness-Related Pathways in Colorectal Cancer Cells

**DOI:** 10.1371/journal.pone.0131363

**Published:** 2015-06-24

**Authors:** Araceli Valverde, Jon Peñarando, Amanda Cañas, Laura M. López-Sánchez, Francisco Conde, Vanessa Hernández, Esther Peralbo, Chary López-Pedrera, Juan de la Haba-Rodríguez, Enrique Aranda, Antonio Rodríguez-Ariza

**Affiliations:** 1 Oncology Department, Maimonides Institute of Biomedical Research (IMIBIC), Reina Sofía Hospital, University of Córdoba, Córdoba, Spain; 2 Spanish Cancer Network (RTICC), Instituto de Salud Carlos III, Madrid, Spain; 3 Research Unit, Maimonides Institute of Biomedical Research (IMIBIC), Reina Sofía Hospital, University of Córdoba, Córdoba, Spain; Southern Illinois University School of Medicine, UNITED STATES

## Abstract

Despite the demonstrated benefits of anti-EGFR/VEGF targeted therapies in metastatic colorectal cancer (mCRC), many patients initially respond, but then show evidence of disease progression. New therapeutic strategies are needed to make the action of available drugs more efficient. Our study aimed to explore whether simultaneous targeting of EGFR/VEGF and cyclooxygenase-2 (COX-2) may aid the treatment and management of mCRC patients. The dual tyrosine kinase inhibitor AEE788 and celecoxib were used to inhibit EGFR/VEGFR and COX-2, respectively, in colorectal cancer cells. COX-2 inhibition with celecoxib augmented the antitumoral and antiangiogenic efficacy of AEE788, as indicated by the inhibition of cell proliferation, induction of apoptosis and G1 cell cycle arrest, down-regulation of VEGF production by cancer cells and reduction of cell migration. These effects were related with a blockade in the EGFR/VEGFR signaling axis. Notably, the combined AEE788/celecoxib treatment prevented β-catenin nuclear accumulation in tumor cells. This effect was associated with a significant downregulation of FOXM1 protein levels and an impairment in the interaction of this transcription factor with β-catenin, which is required for its nuclear localization. Furthermore, the combined treatment also reduced the expression of the stem cell markers Oct 3/4, Nanog, Sox-2 and Snail in cancer cells, and contributed to the diminution of the CSC subpopulation, as indicated by colonosphere formation assays. In conclusion, the combined treatment of AEE788 and celecoxib not only demonstrated enhanced anti-tumoral efficacy in colorectal cancer cells, but also reduced colon CSCs subpopulation by targeting stemness-related pathways. Therefore, the simultaneous targeting of EGFR/VEGF and COX-2 may aid in blocking mCRC progression and improve the efficacy of existing therapies in colorectal cancer.

## Introduction

Colorectal Cancer (CRC) is one of the most commonly diagnosed cancer and cause of cancer mortality in developed countries [1]. In Europe, CRC is the third most common cancer and after lung cancer it was the second most frequent cause of mortality in 2012, with almost 215,000 deaths [2]. Although mortality from CRC has declined slightly during the last two decades, and despite advances in detection and surgical treatment, metastatic CRC (mCRC) is associated with a poor prognosis, with 5-year survival rates in the range of 5% to 8%. Targeting epidermal growth factor receptor (EGFR) has been proven to be an effective therapy in CRC. Particularly, the treatment with monoclonal antibodies (cetuximab or panitumumab) against the extracellular domain of the receptor has become major therapeutic strategies to treat mCRC. However, the responses to EGFR-targeted antibodies are relatively low, with improvements in survival usually lasting only several months, and efficacy limited to certain patient subtypes [3]. In fact, CRC patients defined as "quadruple negative", with tumors lacking mutations in EGFR downstream effectors KRAS, BRAF, PIK3CA, and PTEN, have the highest probability of response to anti-EGFR therapies [4]. On the other hand, different strategies aimed at blocking vascular endothelial growth factor (VEGF) and its receptors have been developed to inhibit angiogenesis in CRC patients [5,6]. Despite the demonstrated benefits of these anti-angiogenic therapies in the management of CRC, many patients with advanced disease will initially respond to anti-VEGF therapy, but then show evidence of disease progression, which suggests resistance to the therapy [7]. Therefore, there is a clear need for better characterization of the processes involved in the inefficacy of anti-EGFR/VEGF targeted therapies and finding new therapeutic strategies to make the action of available drugs more efficient.

During the last decade, it has been shown that in tumors there is a population of cells, commonly referred to as cancer stem cells (CSCs), with ability to proliferate and generate the rest of the tumor mass [8,9]. The ability to self-renewal of CSCs allows homeostasis and maintenance of tumor, in a manner similar as stem cells do in normal tissues. CSCs are much more resistant than differentiated tumor cells to therapies used in clinic [8]. Thus it has been shown that the risk of colorectal cancer recurrence is proportional to the expression in the primary tumor of a series of specific and intestinal stem cells genes that also identify a cell population with CSC properties in the tumor [10]. Importantly, recent studies have shown that CSCs are involved in the mechanisms by which tumors evade both anti-EGFR and anti-VEGF targeted therapies [11,12]

Celecoxib is a selective cyclooxygenase-2 (COX-2) inhibitor, which is used to prevent polyp formation in familial adenomatous polyposis (FAP) patients, a population at high risk for colorectal cancer development [13]. However, studies suggest that celecoxib may have effective anti-tumor and anti-metastatic properties against late stage CRC. Thus, the effectiveness of an antiangiogenic tyrosine kinase inhibitor (axitinib) has been shown to be significantly enhanced when combined with celecoxib in a preclinical model of CRC [14]. Furthermore, the development of multiple kinase inhibitors, has enabled the simultaneous inhibition of EGFR and VEGF pathways. This is the case of AEE788, an oral inhibitor with potent activity against multiple tyrosine kinases including EGFR, and VEGFR [15], which has been shown to exert antitumoral effects in various human cancer models [16–18]. Therefore, the simultaneous targeting of EGFR/VEGF and COX-2 may also aid in blocking mCRC progression. The present study shows that combination of AEE788 with the specific COX-2 inhibitor celecoxib not only demonstrated enhanced anti-tumoral efficacy in colorectal cancer cells but also reduced colon CSCs subpopulation by targeting stemness-related pathways.

## Material and Methods

### Cell culture

Caco-2 cells (ECACC, Salisbury, UK) were grown in MEM with Earle’s salts (PAA Laboratories GmbH, Pasching, Austria) containing 15% fetal bovine serum. HCT-116 cells (DSMZ, Braunschweig, Germany) were grown in McCoy’s 5A medium (Biowest, Nuaillé, France) containing 10% fetal bovine serum (PAA Laboratories). HCT-116 cells harbour an activating mutation in KRAS (G13D), while Caco-2 cells are KRAS wildtype. Culture media were supplemented with 2 mM glutamine, 1% non-essential amino acids, penicillin (100 U/ml), streptomycin (100 μg/ml) and amphotericin B (2.5 μg/ml). Cells were maintained in a humidified atmosphere at 37°C and 5% CO2. After cultures became 80% confluent (usually after 3 days), cells were trypsinized, centrifuged, and suspended in fresh medium. All cells used for experiments displayed > 95% viability and were seeded in 96-well plates, 6-well culture plates, 60-mm culture plates or ultra low-attachment plates (Corning Inc, Lowell, MA, USA). All experiments were carried out in duplicate and repeated at least three times.

### Reagents

AEE788 (Novartis Pharma AG, Basel, Switzerland), NS-398 and celecoxib (Sigma-Aldrich, Madrid, Spain) were dissolved in DMSO to produce stock concentrations of 10 mM, 10 mM and 20 mM, respectively, and stored at -20°C. Working solutions were prepared by diluting thawed stocks into cell culture medium. The concentration of DMSO in the final dilution did not exceed 0.1% v/v. Human EGF was purchased from Santa Cruz Biotechnology (Santa Cruz, CA, USA) and dissolved in glycerol to produce stock concentrations of 10% v/v. Human recombinant VEGF was purchased from Sigma-Aldrich and dissolved in water to produce stock concentrations of 10 μg/mL. For western blot analysis, the following antibodies were used: phospho-EGF Receptor (Tyr1068) rabbit pAb, phospho-VEGF Receptor 2 (Tyr1175)(19A10) rabbit mAb, VEGF Receptor 2 (55B11) rabbit mAb, phospho-p44/42 MAPK (Erk 1/2) (Thr202/Tyr204) (D13.14E) rabbit mAb, phospho-Akt (Thr308)(C31E5E) rabbit mAb, phospho-Akt (Ser473)(587F11) mouse mAb, Akt rabbit pAb and β-Catenin rabbit pAb were purchased from Cell Signaling Technologies (Danvers, MA, USA). EGFR mouse mAb (0.N.268), actin (C-2) mouse mAb, goat anti-rabbit, goat anti-mouse, donkey anti-goat secondary antibodies, and FOXM1 (K-19): sc-500 were purchased from Santa Cruz Biotechnology. MAP kinase ERK1/ERK2 rabbit pAb was from Calbiochem-EMD Millipore (Billerica, MA, USA), COX-2 mouse mAb (Clone CX229) was from Cayman Chemical (Ann Arbor, Michigan, USA) and Ep-Cam mouse mAb was from Chemicon International (Billerica, MA, USA). Propidium iodide was obtained from Sigma-Aldrich and was prepared by dissolving 1 mg in 1 ml phosphate buffered saline. This solution was protected from light and stored at 4°C. RNase, DNase-free was obtained from Roche Applied Science (Indianapolis, IN, USA). Stock concentrations of 500 μg/mL RNase were prepared and kept at– 20°C.

### Cellular proliferation assay

Cell proliferation and viability was assessed using an XTT colorimetric assay (Roche Applied Science). Cells were seeded on 96-well plates at a density of 4,000 cells/well and let to attach for 24 hours. The cells were then treated with AEE788 (0–20 μM) as a single agent or in combination with celecoxib (10 μM) in the presence or absence of EGF (100 ng/mL). Controls cells were treated with the same concentration of the DMSO vehicle. After treatment at indicated times and doses, the XTT assay was performed following the protocol supplied by the manufacturer using a microplate absorbance reader.

### Colonosphere formation assay

For the colonosphere formation assay, after treatments cells were tripsinized, counted and re-seeded at clonal density (1 cell/μl) in 96-well plate with ultra-low attachment surface (Costar, Corning, NY, USA) with serum free Dulbecco’s MEM Nutrient Mixture F+12 Ham medium supplemented with B27 (1:50; Invitrogen, Carlsbad, CA, USA), 10 ng/ml basic fibroblast growth factor (PeproTech, London, UK)), 20 ng/ml EGF (Santa Cruz Biotechnology) and 1% v/v methylcellulose (R&D Systems, Minneapolis, MN, USA) to prevent cell aggregation. The supplements were freshly added every 2–3 days and the number and size of formed colonospheres were evaluated by optical microscopy on day 7 after seeding.

### Western blotting analysis

After treatments cell were harvested with cold PBS and centrifuged at 300 × g, for 5 minutes at 4°C. The cell pellet was incubated for 15 minutes on ice with 1 ml lysis buffer (50 mM Tris-HCl (pH 7.4), 150 mM NaCl, 5 mM ethylenediamine tetraacetic acid (EDTA), 1 mM ethyleneglycol tetraacetic acid (EGTA), 1.5 mM MgCl2, 10% glycerol, 1% NP40, 0.1 M dithiothreitol (DTT), 0.1 M phenylmethylsulfonyl fluoride (PMSF), 1% v/v protease inhibitor cocktail (SERVA, Heidelberg, Germany) and 1% v/v phosphatase inhibitor cocktails 2 and 3 (Sigma-Aldrich) and centrifuged at 10,000 x g for 15 minutes at 4°C. Total protein concentration was quantified by a standard Bradford assay using the colorimetric reagent from BioRad Laboratories (Hercules, CA, USA).

Proteins (12.5 μg) were separated onto SDS polyacrylamide gels using a 4–12% Bis-Tris gradient gels in the BioRad Criterion System and transferred to nitrocellulose membranes, which were blocked with 3% BSA, and probed with the appropriate antibodies. Immunocomplexes were detected with appropriate horseradish peroxidase-conjugated secondary antibodies and detected by enhanced chemiluminescence with the ECL Plus Western Blotting Detection System or ECL Advance Western Blotting Detection Kit (GE Healthcare Life Sciences, Little Chal-font, UK). Images were captured on a ChemiDoc XRS Imaging System (BioRad Hercules, CA, USA).

### Combined annexin-V/propidium iodide staining

The fraction of apoptotic cells was estimated after 48h of treatment of cells growing in the absence or in the presence of EGF (100 ng/mL) with different doses of AEE788 and/or celecoxib in 6-well plates at a density of 3 × 10^6^ cells/well. Viability was assessed by using an Annexin-V/propidium iodide (PI) staining kit (Bender MedSystems, Vienna, Austria), according to manufacturer´s recommendations. Binding of fluorescein-conjugated Annexin-V and PI was measured by flow cytometry (FACSCalibur; BD, Franklin Lakes, NJ, USA) to quantify the percentage of apoptotic cells.

### Enzyme-linked immunosorbent assays (ELISA) for analyses of VEGF and prostaglandin E2 production

VEGF production by cells was estimated by ELISA assays (Human VEGF-A Platinum ELISA e-Bioscience) performed on cell culture supernatants 48h after the different treatments and following the protocol supplied by the manufacturer. In brief, cultures were centrifuged at 300 × g for 5 minutes at 4°C and supernatants were collected, aliquoted, and stored at -80°C until assay. Optical density was measured at 450 nm with the correction wavelength set at 650 nm, using a microplate reader. Prostaglandin E2 (PGE2) production by cells was estimated using the PGE2 EIA kit, (Enzo Life Sciences, Farmingdale, NY, USA). This assay was performed on cell lysates 12 h after the treatments with AEE788 and/or Celecoxib and following the protocol supplied by the manufacturer. Optical density was measured at 450 nm with the correction wavelength set at 570–590 nm, using a microplate reader.

### Cell cycle analysis

Cells (0.5–1 x 10^6^ cells) were trypsinized and resuspended in PBS. Ice-cold 100% ethanol was added in a drop-wise manner while gently vortexing and incubated for 20 minutes at room temperature. Samples were centrifuged at 300 × g for 5 minutes, resuspended in PBS containing 50 μg/ml propidium iodide plus 100 μg/ml RNase A and incubated for 20 minutes at room temperature protected from light. Analysis and measurement of propidium iodide fluorescence were performed on a FACSCalibur (BD Biosciences) flow cytometer (FACS; BD, Franklin Lakes, NJ, USA).

### Angiogenesis assay

Angiogenesis was measured as the ability of endothelial cells to form three-dimensional structures in matrigel basement membrane matrix (BD Biosciences) *in vitro*. For angiogenesis assay, HUVECs cells (CC-2519 Lonza Sales AG, Basel, Switzerland) were cultured on top of the matrigel at a density of 20,000 cells/well in Caco-2-conditioned media after different treatments. Caco-2-conditioned media after different treatments was concentrated in Amicon Ultra-0,5 Centrifugal Filter Devices (EMD Millipore) with the capability for obtaining high concentration factors and removing drug residues. As control, HUVECs cells were cultured in EBM medium (Lonza Walkersville, MD USA) in the presence or the absence of VEGF (10 μg/ml). HUVECs were incubated for 24 hours at 37°C in 5% CO_2_ atmosphere and following staining with the fluorescence dye Calcein AM (Cell Biolabs, INC), the tube formation was examined and photographed using a fluorescence microscope. The extent of tube formation (average tube length and branch points) was quantified through imaging software (Image J software).

### Antibody arrays

Specific antibody arrays were used to determine the expression of intracellular receptor tyrosine kinase (RTK)-related signaling pathways and the expression of pluripotency-related proteins in whole-cell extracts following the protocols supplied by the manufacturers. In brief, cellular extracts were diluted and incubated overnight at 4°C with the PathScan RTK Signaling Antibody Array Kit Cell Signaling Technology) or the Proteome Profiler Human Pluripotent Stem cell Array (Cat#ARY010, R&D Systems) followed by a biotinylated detection antibody cocktail. Streptavidin-conjugated HRP and LumiGLO Reagent were then used to visualize the bound detection antibody by chemiluminescence. An image of the slide was captured with a digital imaging system (ImageQuant LAS 4000, GE Healthcare Life Sciences, Pittsburgh, PA, USA) and was analyzed using the analysis software provided for measuring the spot relative intensities.

### Scratch-wound healing assay

Cells were grown on 6 multi-wells plates to a nearly confluent monolayer. After incubation for 24h the confluent monolayers were scratched to form a “wound” using a sterile needle. Cellular debris were removed by washing with PBS. The cells were then cultured under the different treatments in the appropriate media for 24 h. The images were recorded at 0 and 24 h to monitor the migration of cells into the wounded area using a light photomicroscope. To quantify (Image-J software) the percentage of wound (scratch area) at 0 h (control) was arbitrarily assigned as 100% and the percentage of wound healing at 24h (untreated control and treated) was compared to control. Each assay was performed in triplicate.

### Quantitative real-time reverse transcriptase-polymerase chain reaction

Total RNA was extracted by RNeasy Plus Mini Kit (Quiagen), according to manufacturer’s recommendations. RNA concentration was determined spectrophotometrically at 260 and 280 nm and RNA samples were stored at -80°C until use. One-step reverse transcriptase-polymerase chain reaction (RT-PCR) was performed using the QuantiTect SYBR Green RT-PCR kit (QIAGEN GmbH, Hilden, Germany) following manufacturer’s protocol. Expression levels of VEGF gene were measured by quantitative real-time RT-PCR using the LightCycler thermal cycler system (Roche Diagnostics, Indianapolis, IN, USA). Glyceraldehyde phosphate dehydrogenase (GAPDH) was used as housekeeping gene. Theoretical size of PCR products, sequence of primers used for study and efficiency were: VEGF: (254 bp), Forward primer: 5’-CCCTGATGAGATCGAGTACATCTT -3’; Reverse primer: 5’-AGCAAGGCCCACAGGGATTT-3, Efficiency: 2; GAPDH: (240 bp), Forward primer: 5’-TGATGACATCAAGAAGGTGGTGAAG-3’;

Reverse primer: 5’-TCCTTGGAGGCCATGTAGGCCAT-3’, Efficiency: 2; Results were normalized to that of GAPDH and quantification of relative expression was determined by the 2^-ΔΔCt^ method.

### Transient expression of mutant K-RAS gene in Caco-2 cells

Caco-2 cells were transiently transfected with pBabe K-Ras 12V plasmid or the empty vector (pBABE-puro), provided by Addgene (plasmids #12544 and #1764). PureYield Plasmid Midiprep System (Promega) was used following the protocol supplied by the manufacturer. Cells were seeded at 90% confluency in 6‐well plates and after 24 h cells were transfected using Lipofectamine 3000 (Life Technologies) as transfection reagent, following the manufacturer’s instructions. Analysis of EGF-independent activation of ERK1/2 by western blot and cell proliferation assays were performed in transfected cells.

### Immunofluorescence confocal microscopy

Cells were grown on poly-L-Lysine-treated coverslips to form a nearly confluent monolayer and after 6h of the different treatments. Then, the culture media was discarded, cells were washed thrice in PBS and permeabilized in methanol. After washing with PBS, coverslips were incubated with Anti-β-Catenin (1/250) mouse mAb (BD) and FOXM1 (1/250) rabbit pAb (Santa Cruz Biotechnology), for 1h at room temperature, washed thrice in PBS and labeled with an anti-mouse IgG alexa fluor 488-labeled antibody (1/500) (Molecular Probes, Eugene, OR, USA) and with anti-rabbit IgG (H+L) alexa fluor 594-labeled antibody (1/500) (Santa Cruz Biotechnology), for 1h at room temperature. Finally, cells were incubated with 300 nM 4’,6-diamidino-2-phenylindole dihydrochloride (DAPI) in PBS for 5 minutes at room temperature. Coverslip were mounted using Aqua Poly/Mount (Polysciences, Warrington, PA, USA) and visualized in a Zeis LSM 5 Exciter Confocal laser-scanning microscope (Zeiss, Germany). Images were analyzed using the image-J software (National Institutes of Health).

### Co-immunoprecipitation assays

Cells were lysed in co-IP buffer (10 mM HEPES, 10 mM KCl, 1.5 mM MgCl_2_, 0.5 mM DTT, 0.5 mM, PMSF, 0.075% NP40 and 1% v/v proteases/phosphatases inhibitor cocktails), centrifuged and cleared by incubation with 22.5 μl of Protein A/G gel for 1 h at 4°C. The pre-cleared supernatant was subjected to IP using the indicated first antibodies at 4°C for 1 h. Then, the protein complexes were collected by incubation with 22.5 μl of Protein A/G gel at 4°C overnight. The collected protein complexes were washed thrice with PBS buffer, centrifuged at 14000 x g, 4°C for 10 min, and analyzed by western blotting.

### Statistical analysis

All data are expressed as mean ± standard error of mean. All Statistical analyses were performed using GraphPad Prism 5. Before comparing two data groups, a normality test and an equal variance test were performed. If data groups passed both tests, a comparison was made by a parametric approach (unpaired Student’s t-test). If the normality and/or equal variance test was violated, a comparison was made by a nonparametric method (Mann-Whitney test). Differences were considered statistically significant at p < 0.05.

## Results

### AEE788 inhibits cell proliferation, induces apoptosis and alters cell cycle in colorectal cancer cells

**Fig 1:**
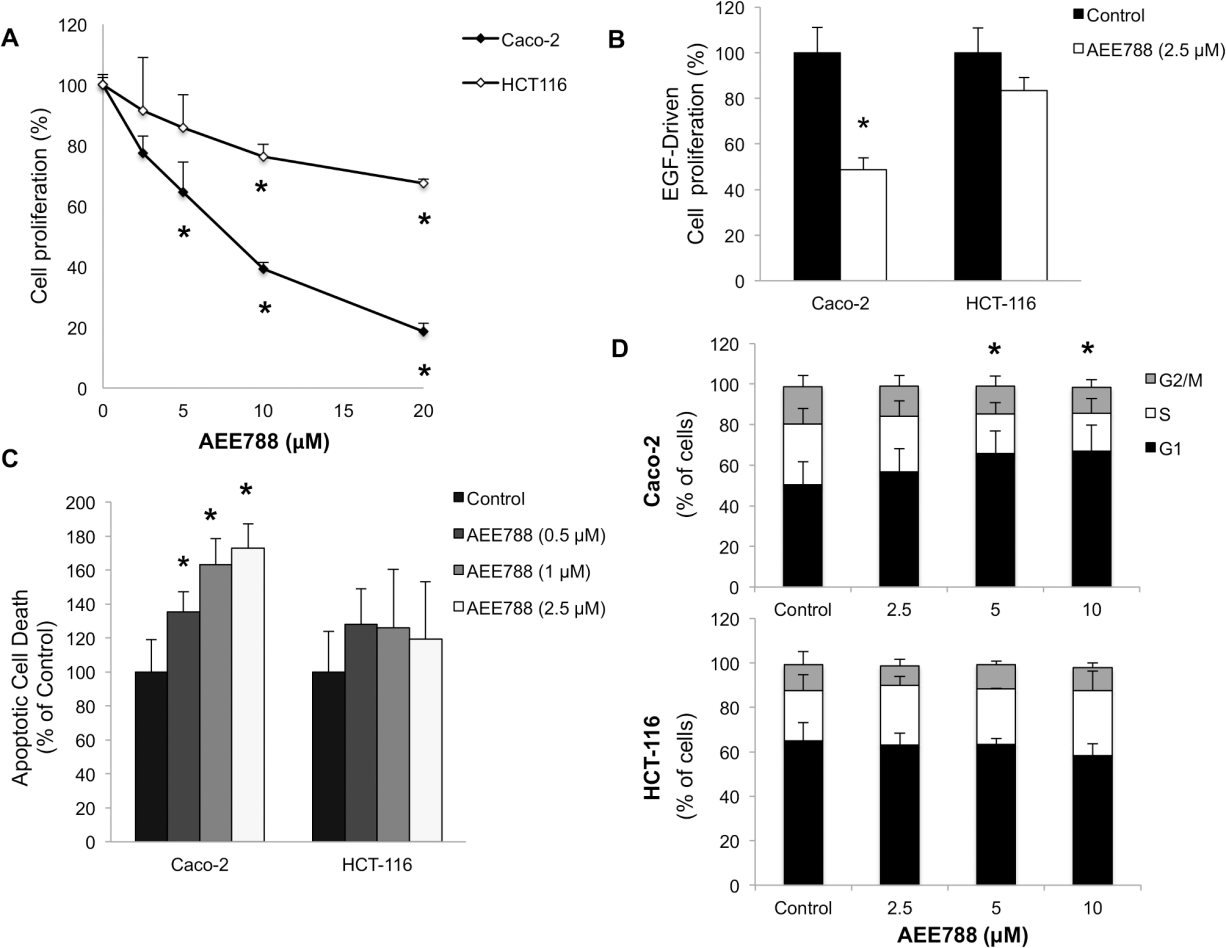
AEE788 inhibits cell proliferation, induces apoptosis and alters cell cycle in colorectal cancer cells. A) Cell proliferation was evaluated after 72h of treatment with different doses of AEE788. B) Inhibition of cell proliferation by AEE788 was tested in cells growing in the presence of EGF (100 ng/ml). C) The fraction of apoptotic cells was estimated after 48 h of treatment with different doses of AEE788 of cells growing in the presence of EGF (100 ng/mL). D) Analysis of cell cycle was performed by flow cytometry after 48 h of treatment with different doses of AEE788 of cells growing in the presence of EGF (100 ng/mL). Data are means ± SEM of three independent experiments (*p <0.05, compared with the control).

AEE788 exerted an antiproliferative effect in HCT-116 and Caco-2 cells with a greater antitumor effect in the case of Caco-2 cells, (Fig 1A). The different sensitivity to AE788 between the two cell lines was more evident in the case of EGF-driven cell proliferation, in which 2.5 µM AE788 reduced proliferation of Caco-2 cells by 60%, whereas K-Ras mutated HCT-116 cells were not significantly affected (Fig 1B). In agreement with these results, AEE788 exerted a dose-dependent apoptotic cell death in Caco-2 but not in HCT-116 cells (Fig 1C). Moreover, the treatment of Caco-2 cells with different doses of AEE788 resulted in an increased percentage of cells in G1 phase in a dose-dependent manner, compared to untreated cells, while these alterations in cell cycle were not observed in AE788-treated K-Ras mutated HCT-116 cells (Fig 1D).

### AEE788 inhibits EGFR signaling in colorectal cancer cells

**Fig 2:**
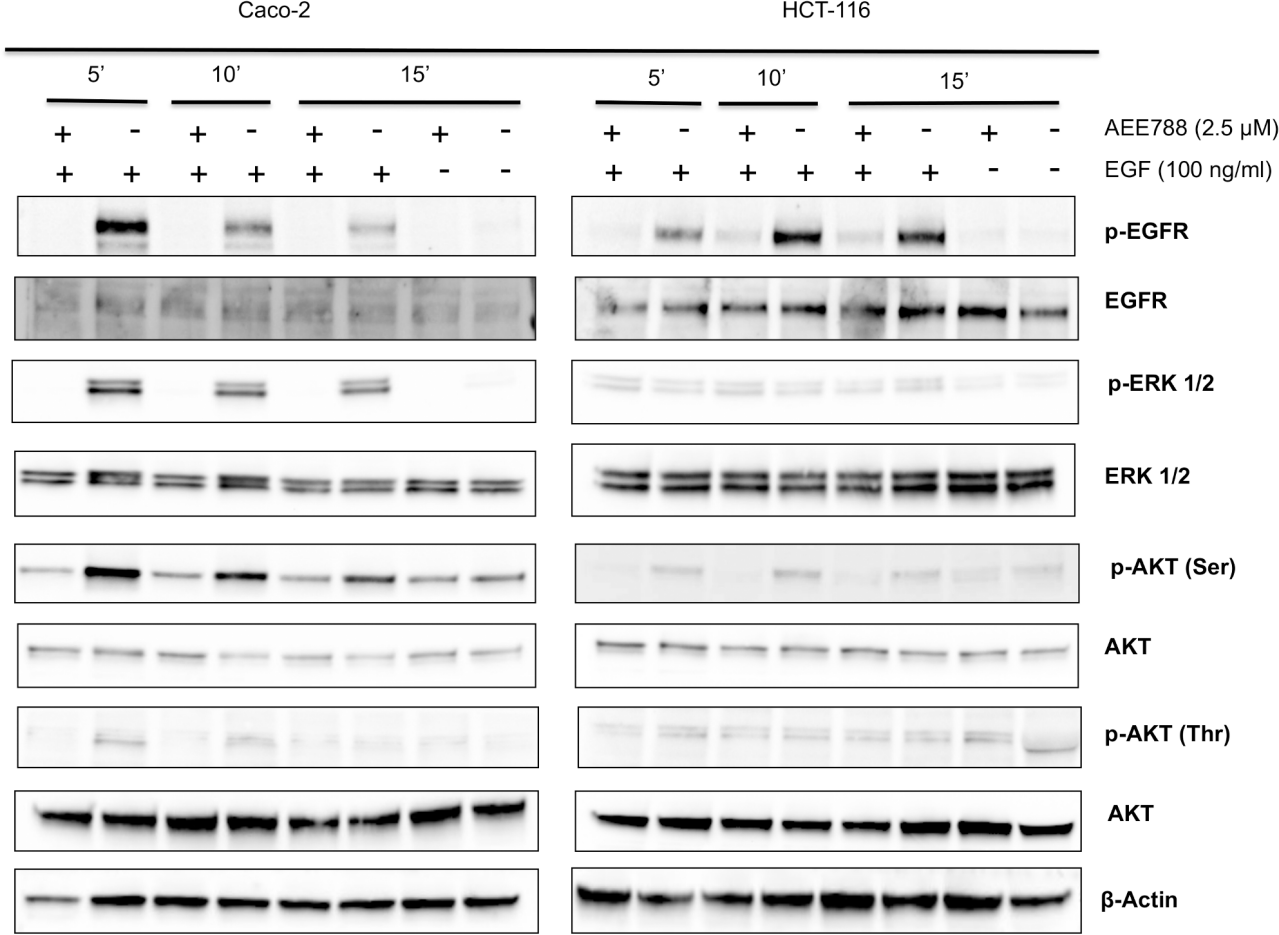
AEE788 inhibits EGFR signaling in colorectal cancer cells. The phosphorylated and non-phosphorylated forms of EGFR, ERK 1/2 and Akt were detected by Western-blot using specific antibodies. Cells were grown in the absence or presence of EGF (100 ng/mL) and treated with AEE788 (2.5 µM) for 5, 10 or 15 min. The expression level of -actin was included as loading control.

We next analyzed the activation of EGFR, ERK and AKT kinases to evaluate the significance of the EGFR signal blockade in the anti-tumoral effects of AEE788 (Fig 2). The treatment with AEE788 inhibited EGFR phosphorylation in both HCT-116 and Caco-2 cells. However AEE788 effectively inhibited EGF signaling axis only in Caco-2 cells, as indicated by the inhibition of EGF-induced phosphorylation of ERK 1/2 (Fig 2). Also, an inhibition of EGF-induced phosphorylation of Akt Thr was observed in AEE788-treated Caco-2 but not in HCT-116 cells. Therefore, the observed anti-EGFR activity of AEE788 in colon cancer cells is strongly dependent on wild-type K-Ras status.

### AEE788 inhibits the VEGF-driven proliferation in Caco-2 cells

In addition to anti-EGFR activity, AEE788 also targets VEGFR signaling. In this regard, the function of VEGF is not limited to angiogenesis and vascular permeability, and it is known that both autocrine and paracrine VEGF signalling occur in tumour cells [19]. Therefore, we next investigated whether AEE788 impair VEGF signaling in colon cancer cells. As shown in S1 Fig, the treatment with AEE788 inhibited VEGF-driven cell proliferation in Caco-2 but not in HCT-116 cells.

### COX-2 inhibition augments the antitumoral efficacy of AEE788

**Fig 3:**
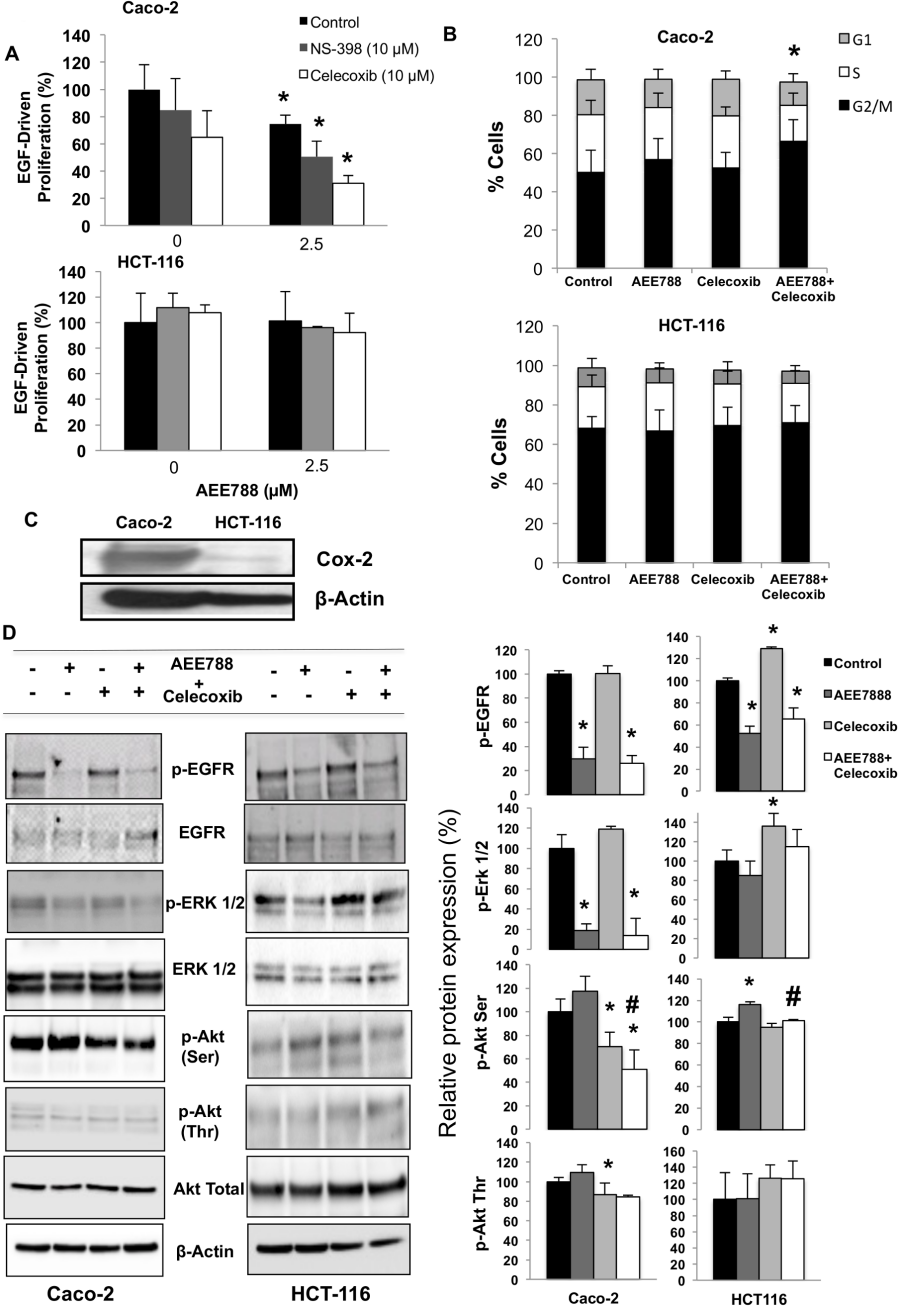
Inhibition of COX-2 enhances the antitumor efficacy of AEE788 in colorectal cancer cells. A) EGF-driven cell proliferation was assayed in cells growing in the presence of EGF (100 ng/ml) and in the presence or absence of AEE788 (2.5 μM), celecoxib (10 μM) and NS-398 (10 μM). B) Analysis of cell cycle was performed by flow cytometry after 48 h of treatment with AEE788 (2.5 μM) and/or celecoxib (10 μM) of cells growing in the presence of EGF (100 ng/mL). C) Expression levels of cyclooxygenase-2 (COX-2) were analyzed by western-blot in whole cell extracts form Caco-2 and HCT-116 cells. Expression of β-actin is included as loading control. D) The phosphorylated and non-phosphorylated forms of EGFR, ERK 1/2 and Akt were detected by Western-blot using specific antibodies. Cells were grown in the presence of EGF (100 ng/mL) and treated with AEE788 (2.5 µM) and/or celecoxib (10 μM) for 6h. The expression level of -actin was included as loading control. The corresponding densitometric analysis is also shown. Data are means ± SEM of three independent experiments (*p <0.05, compared with the control; # p<0.05, compared with AEE788-treated cells).

It is known that VEGF stimulates COX-2 expression and prostaglandin synthesis, which in turn induces VEGF production [20,21]. Therefore, the combination with COX-2 inhibitors can be an efficient way to increase the activity of AEE788. As shown in Fig 3, the addition of both NS-398 and celecoxib, two well-known COX-2 selective inhibitors, enhanced the anti-tumoral effect of AEE788, as indicated by the inhibition of cell proliferation (Fig 3A). Furthermore, the combined treatment with AEE788 and celecoxib increased the percentage of Caco-2 cells arrested in G1 (Fig 3B). In particular, a significantly higher percentage of cells in G1 phase was observed following the combined treatment than with either drugs alone. On the contrary, none of these effects were observed in HCT-116 cells, which can be related to the much lower expression levels of COX-2 in these cells compared to Caco-2 (Fig 3C) and their mutated K-Ras status. Thus, the transfection of Caco-2 cells with a mutant K-Ras expressing plasmid confirmed that downstream activation of the EGFR-Ras-ERK pathway, as shown by the EGF-independent ERK1/2 phosphorylation, abolished the antiproliferative activity of AEE788 and/or celecoxib (S2 Fig). Furthermore, celecoxib inhibited by more than 70% PGE2 production in Caco-2 cells (S3 Fig). Moreover, it was found that AEE788 treatment decreased COX-2 protein expression in Caco-2 cells, explaining the 40% reduction in PGE2 production, while the inhibition of COX-2 enzymatic activity with celecoxib did not affect COX-2 expression (S3 Fig). Accordingly, the combined treatment of AE788 and celecoxib inhibited EGFR, ERK 1/2, and AKT (Ser/Thr) phosphorylation in Caco-2 cells (Fig 3D). Quantitative analysis using an antibody panel against signaling kinases showed that the combined treatment of Caco-2 cells with AEE788 and celecoxib caused a significantly higher inhibition of VEGFR, Akt (Ser/Thr) and Stat3 phosphorylation than with either drug alone (S4 Fig).

### COX-2 inhibition intensifies the anti-angiogenic activity of AEE788

**Fig 4:**
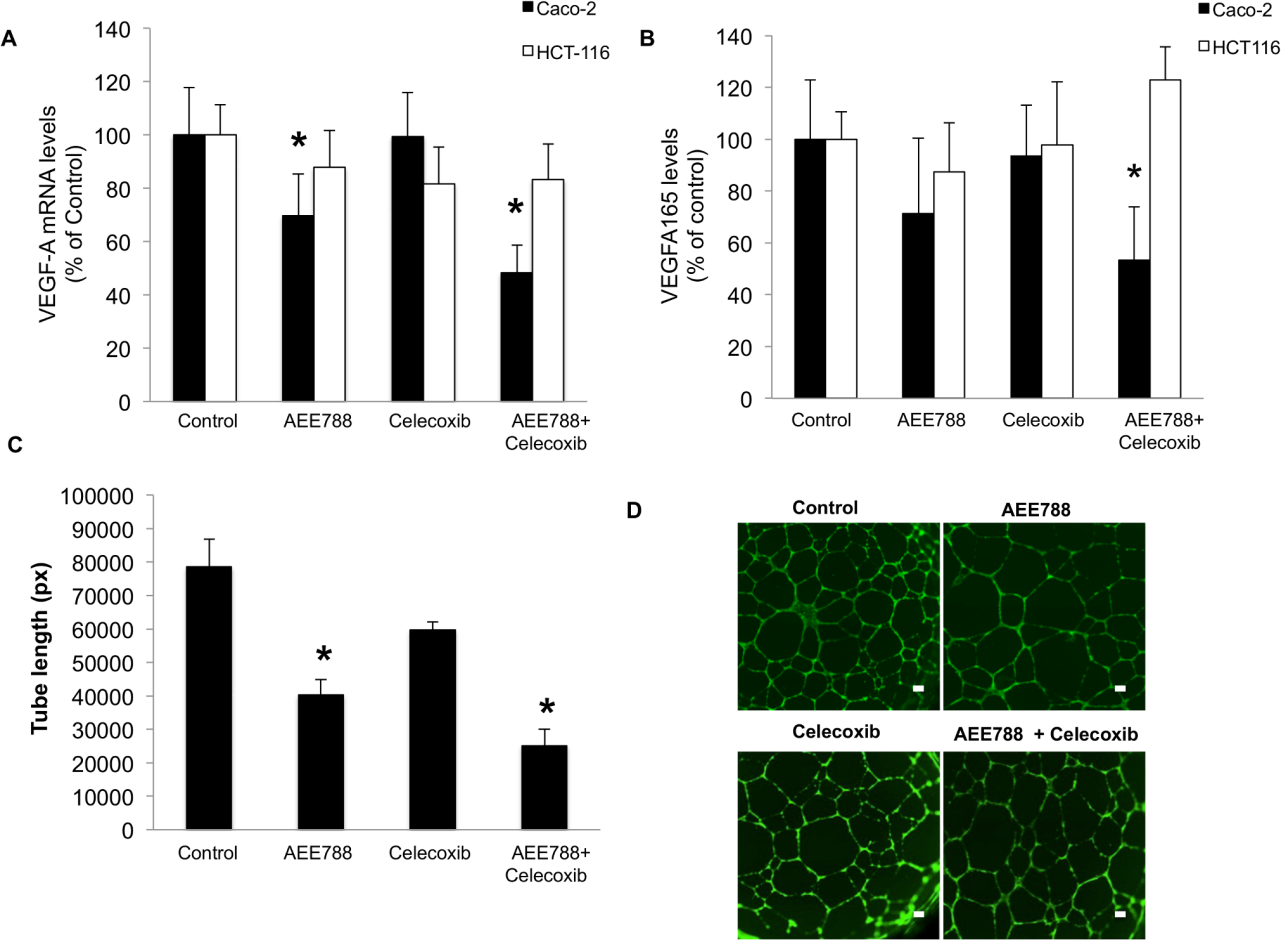
Celecoxib intensifies the anti-angiogenic activity of AEE788. A) VEGF-A mRNA expression levels were assayed by real time RT-PCR in colorectal cancer cells growing in the presence of EGF (100 ng/ml) and exposed to the indicated treatments for 48 h. B) VEGFA165 levels were quantified by ELISA in the conditioned media collected from cells growing in the presence of EGF (100 ng/ml) and treated with AEE788 (2.5 µM) and/or celecoxib (10 μM) for 48h. Data are means ± SEM of three independent experiments (*p <0.05, compared with the control). C) The angiogenic activity of media conditioned by Caco-2 cells exposed for 24 h to the indicated treatments was evaluated using the endothelial tube assay as described under Material and Methods. Data are the total length of formed tubes in pixels (px), showing means ± SEM of three independent experiments (*p <0.05, compared with the control). D) Representative images of the formed interconnected networks after the treatment of endothelial cells with the indicated Caco-2 cells conditioned media. The extent of tube formation was quantified as indicated in the Material and Methods section. (Final magnification: X40, scale bar corresponds to 100 microns).

The inhibition of COX-2 in combination with the anti-EGFR/VEGFR activity of AEE788, caused a significant reduction in the VEGF-A mRNA expression (Fig 4A) and VEGF165 protein secretion levels (Fig 4B) in Caco-2 but not in HCT-116 cells. Moreover, endothelial tube formation assays showed that the angiogenic activity of Caco-2 conditioned medium was significantly lower when cells were treated with AEE788 and celecoxib than with either drug alone. (Fig 4C and 4D).

**Fig 5:**
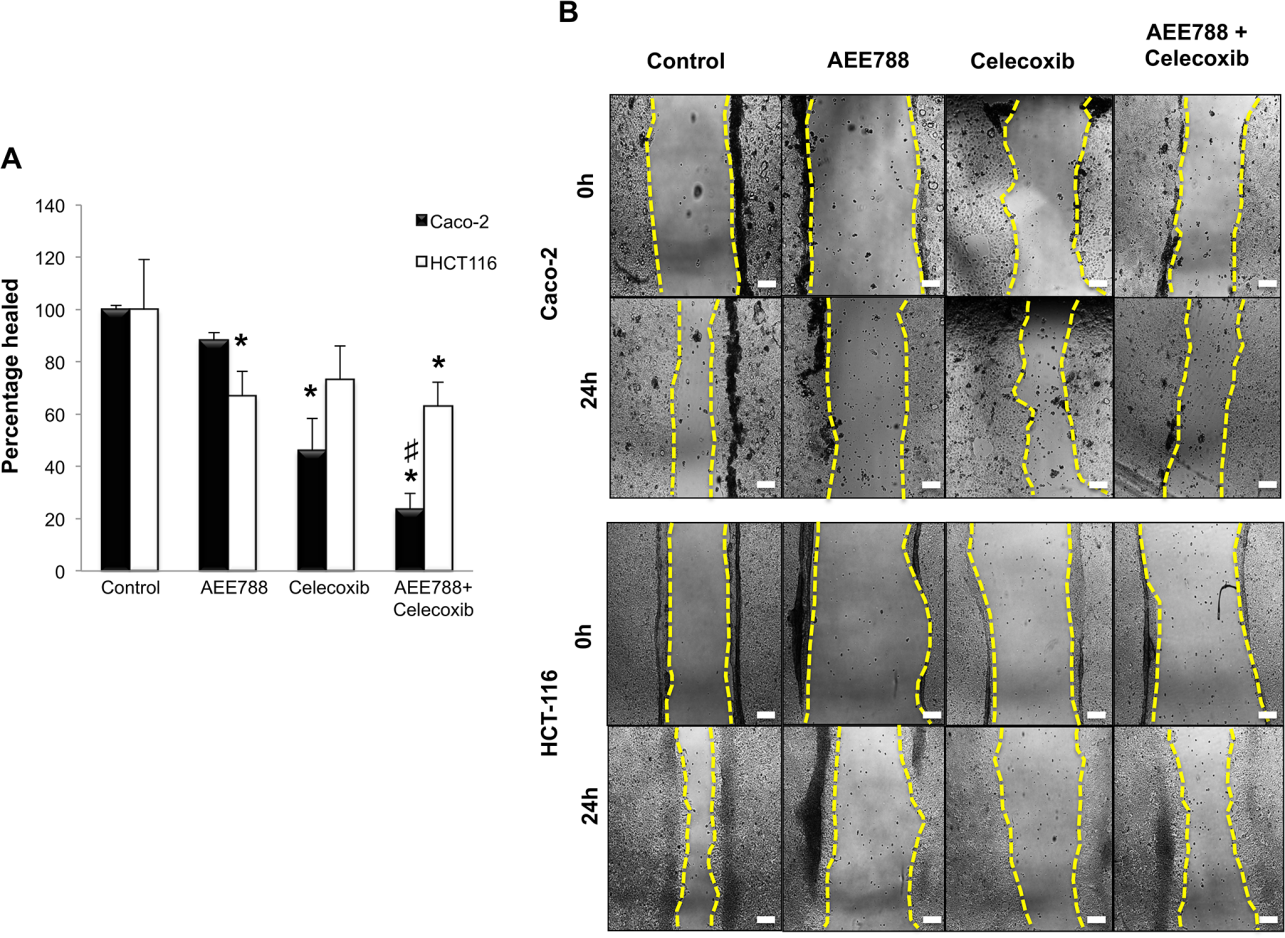
Combined AE788/Celecoxib treatment reduces the migratory capacity of colorectal cancer cells. A) Scratch wound healing assay was used to analyze the inhibition of cell migration in colorectal cancer cells treated for 24 h with AEE788 (2.5 µM) and/or celecoxib (10 µM). Data are means ± SEM of three independent experiments (*p <0.05, compared with the control; # p<0.05, compared with AEE788-treated cells). Final magnification: X100, scale bar corresponds to 100 microns. B) Representative images of scratched areas in confluent Caco-2 and HCT-116 cell layers. The yellow lines indicate the invasive front in the wound healing assay. Wound closure was photographed at 0h and 24 h after wounding. The scratched area at control (0 h) was arbitrarily assigned as 100%.

In addition to the functions of VEGF in angiogenesis and in endothelial cells, the promotion of cancer cell migration has been shown to be among the autocrine functions of VEGF on tumor cells [22,23]. Therefore, we next investigated the effects of AEE788 and/or celecoxib on Caco-2 and HCT-116 cells migration using the scratch-wound healing assay. As shown in Fig 5, the combined treatment was the most effective in reducing the migration of Caco-2 cells (Fig 5A). On the contrary, none of the treatments showed great efficacy in reducing the migratory capacity of HCT-116 cells (Fig 5B).

### The combined AEE788/celecoxib treatment alters subcellular distribution of β-catenin in Caco-2 cells

**Fig 6:**
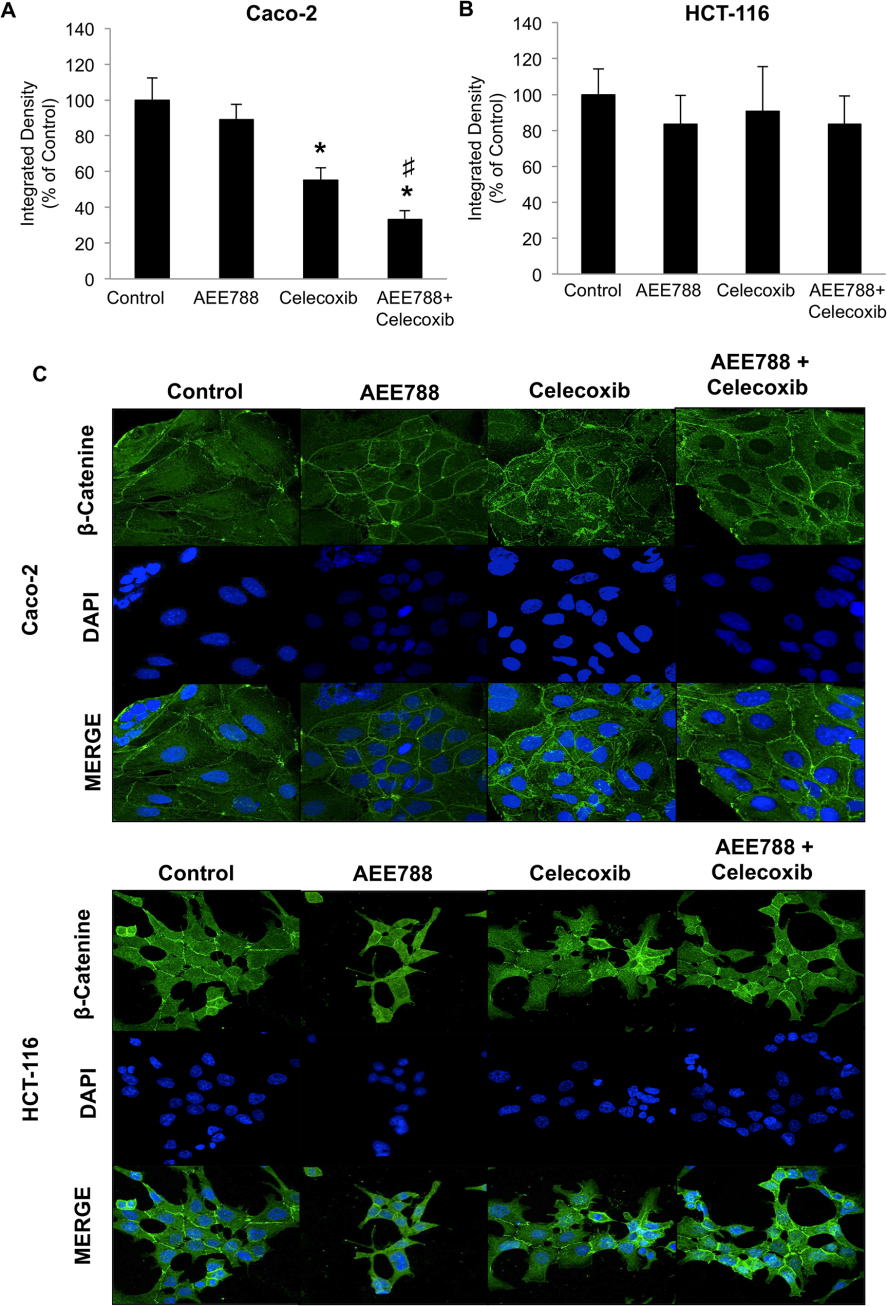
Combined AEE788/Celecoxib treatment alters subcellular distribution of β-catenin in Caco-2 cells. To determine subcellular localization of β-catenin, both Caco-2 (A) and HCT-116 (B) cells were treated with AEE788 (2.5 µM) and/or celecoxib (10 μM) for 6 h, stained for β-catenin immunofluorescence (green) and counterstained with DAPI (blue). Merged images of β-catenin and DAPI staining are also shown. Final magnification: X400. Nuclear β-catenin levels were quantified as the integrated density of β-catenin signal in confocal microscopy images using the Image-J software. Data are means ± SEM of three independent experiments (*p <0.05, compared with the control; # p<0.05, compared with AEE788-treated cells).

It is well known the crucial role for hyperactivated Wnt–β-catenin signaling in colorectal cancer. Both β-catenin (CTNNB1) and adenomatous polyposis coli (APC), which is a negative regulator of β-catenin stability, are frequently mutated genes in colorectal tumors [24]. Hence, Caco-2 cells harbor an APC mutation, whereas HCT-116 cells harbor a single amino acid deletion (CTNNB1 p.S45del) which prevent β-catenin phosphorylation and degradation by the proteasome. Both mutations result in the accumulation of β-catenin, which then enters the nucleus and cause aberrant activation Wnt–β-catenin-regulated genes. Therefore we next explored whether the treatment with AE788 and/or celecoxib modify the subcellular distribution of β-catenin in colon cancer cells. As shown in Fig 6, confocal microscopy analyses demonstrated that both Caco-2 and HCT-116 expressed high levels of β-catenin. In untreated cells, β-catenin displayed membrane associated, cytosolic and nuclear localizations. Notably, the combined AEE788/celecoxib treatment drastically reduced nuclear β-catenin levels in Caco-2 cells (Fig 6A). However, none of the treatments significantly altered the subcellular distribution of β-catenin in HCT-116 cells (Fig 6B).

### The combined AEE788/celecoxib treatment impairs FOXM1- β-catenin interaction in Caco-2 cells

**Fig 7:**
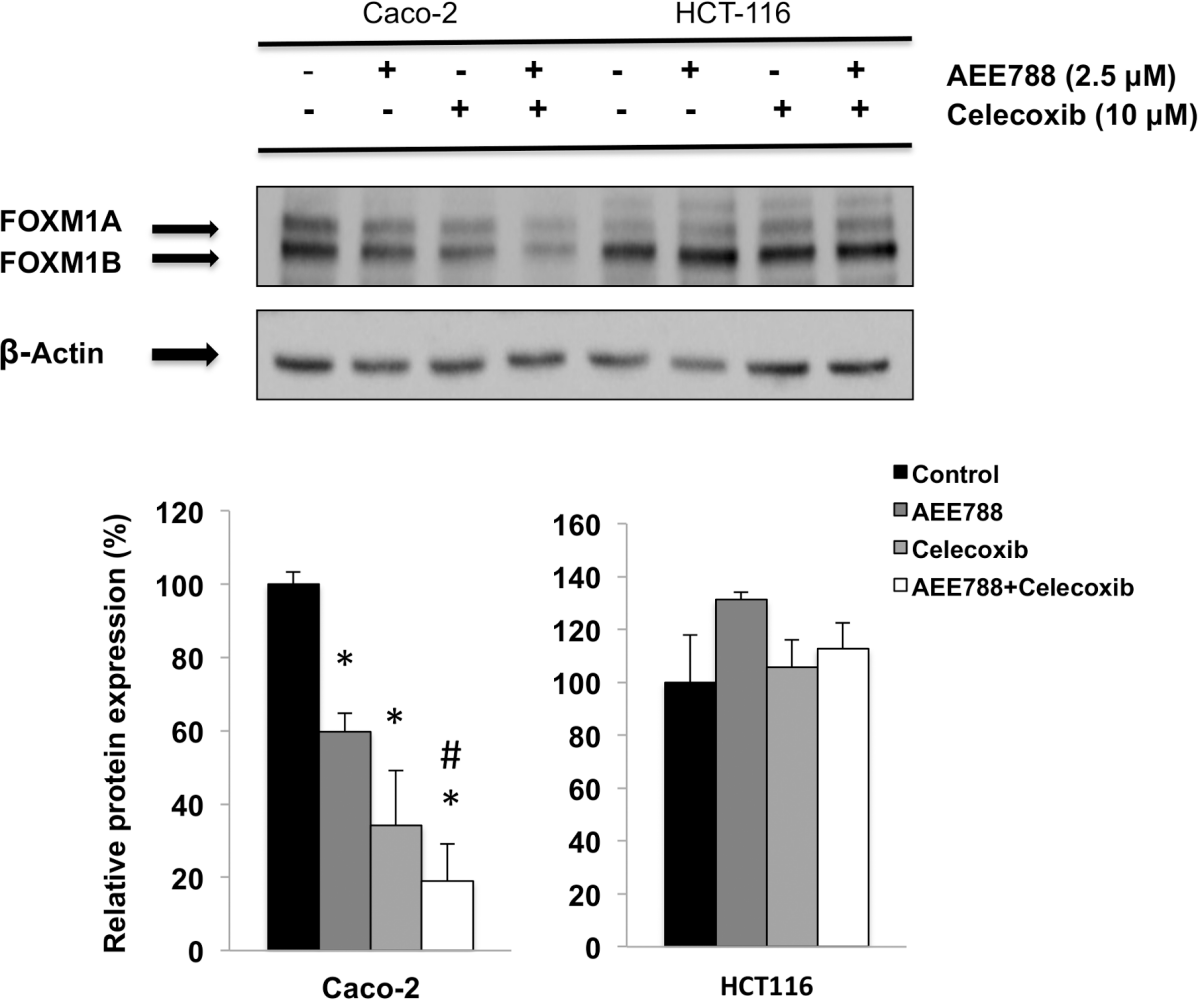
Combined AEE788/Celecoxib treatment downregulates FOXM1 protein levels in colorectal cancer cells. FOXM1 expression was analized by western-blot in cells were grown in the presence of EGF (100 ng/mL) and treated with AEE788 (2.5 µM) and/or celecoxib (10 μM) for 6h. The expression -actin is included as loading control. The corresponding densitometric analysis is also shown. Data are means ± SEM of three independent experiments (*p <0.05, compared with the control; # p<0.05, compared with AEE788-treated cells).

**Fig 8:**
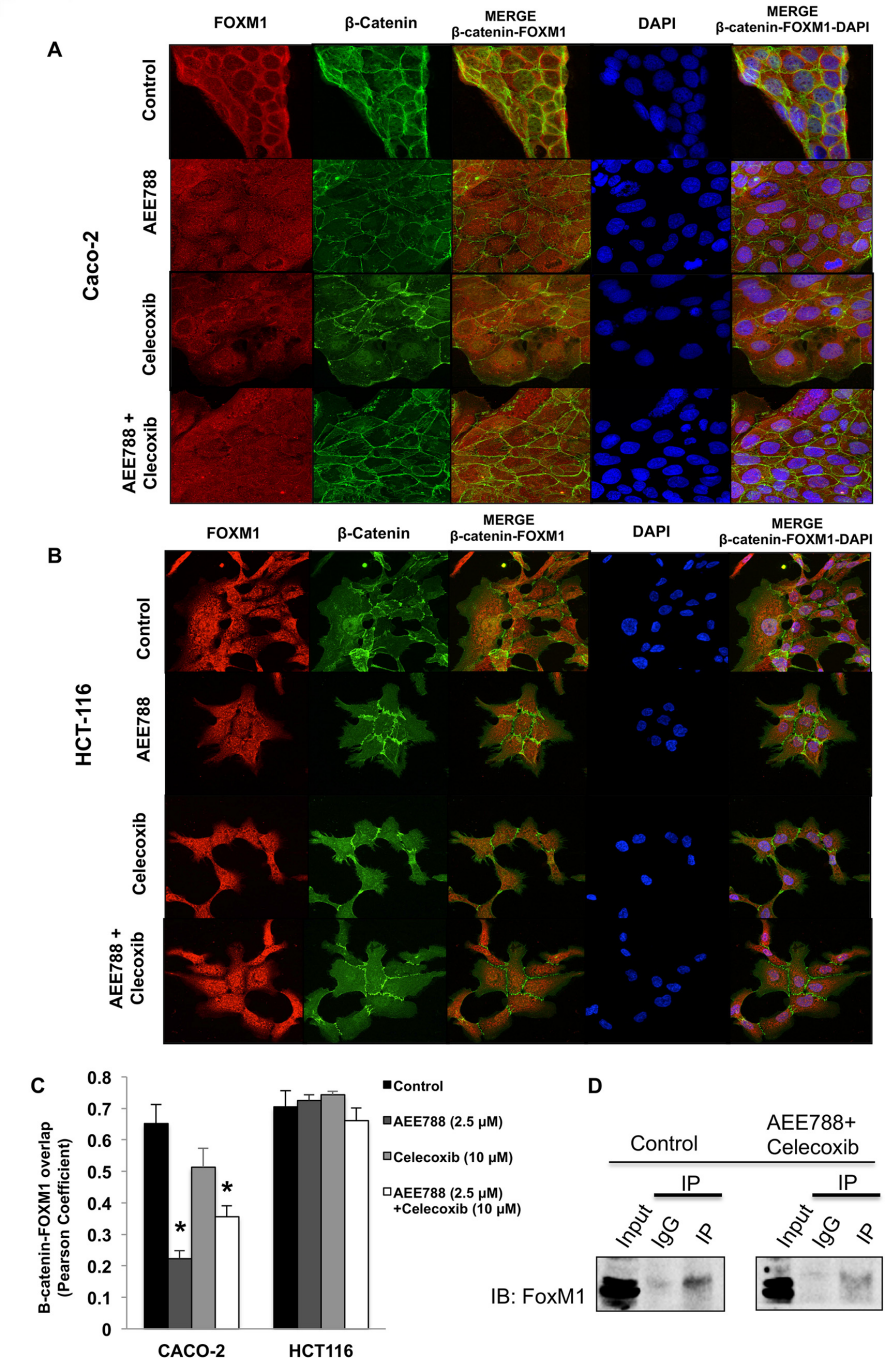
Combined AEE788/Celecoxib impairs FOXM1- β-catenin interaction. To determine subcellular localization of β-catenin and FOXM1, both Caco-2 (A) and HCT-116 (B) cells were exposed to AEE788 (2.5 βM) and/or celecoxib (10 μM) for 6 h, stained for β-catenin (green) and FoxM (red) immunofluorescence, and counterstained with DAPI (blue). Merged images of β-catenin, FOXM1 and DAPI staining are also shown. Final magnification: X400. C) Pearson´s coefficient analysis was performed for the co-localization in cell nuclei of β-catenin and FOXM1. Data are means ± SEM of three independent experiments (*p <0.05, compared with the control). D) Cell extracts of Caco-2 cells after 6 h of the indicated treatments were subjected to IP using β-catenin antibody or control IgG, followed by IB with FOXM1 antibody.

The molecular mechanisms governing β-catenin nuclear import during Wnt signaling are poorly understood. Remarkably, β-catenin lacks a nuclear localization signal and does not depend on importins or the small Ras-related GTPase Ran for nuclear translocation [25]. However, a recent study has demonstrated that nuclear translocation of β-catenin depends on its binding to the forkhead box M1 (FOXM1) transcription factor [26]. Therefore, we next explored whether the expression and subcellular location of FOXM1 were altered in our experimental conditions. As shown in Fig 7, the treatment with AEE788, celecoxib, and especially the combined treatment, significantly downregulated the FOXM1 protein levels of Caco-2 cells. On the contrary, none of the different treatments exerted a significant effect on FOXM1 expression in HCT-116 cells. Besides, confocal microscopy analysis in Caco-2 cells (Fig 8A) and HCT-116 (Fig 8B), revealed that FOXM1 and β-catenin co-localization in cell nuclei was significantly lower when Caco-2 cells (Fig 8C) were treated with AEE788 or the combined treatment. However, neither AEE788 nor celecoxib, alone or in combination, significantly altered FOXM1 and β-catenin co-localization in HCT-116 cells. The direct interaction of FOXM1 and β-catenin was also determined by co-immunoprecipitation assays. As shown in Fig 8D, FOXM1 could be detected in the β-catenin precipitates, and its level was reduced upon treatment of Caco-2 cells with AEE788 and celecoxib, suggesting that the combined treatment impairs the interaction of both proteins.

### The combined AEE788/celecoxib treatment targets stemness-related pathways in colorectal cancer cells

Numerous studies indicate that Wnt—β-catenin signaling contributes to cancer progression through the maintenance of highly tumorigenic subpopulations of cancer cells termed cancer stem cells (CSCs) [27–29]. Among the methods used for the study of CSCs are functional assays of self-renewal capacity, such as tumorosphere formation *in vitro*. In this assay, cells are cultured at clonal density with serum free medium in low-adherence plates. Under these conditions, only a subpopulation of tumor undifferentiated cells with stem characteristics (CSCs) survives, which is capable of generating tumorospheres (colonospheres) in suspension by self-renewal. Therefore, we next analyzed the impact of AEE788/celecoxib treatment on the capability of colorectal cancer cells to form colonospheres *in vitro*. Firstly, to verify that individual colonospheres were derived from single cells, control experiments were performed using lipophylic fluorescent stains. Thus, when equal numbers of DiI (Red)- or DiO (Green)-labelled cells were mixed prior to performing the colonosphere formation assay, formed spheres contained only one or the other label (S5 Fig). Furthermore, Caco-2 and HCT116 cells growing as colonospheres displayed higher expression levels of β-Catenin, Oct 3/4, Nanog, Sox-2 and Ep-Cam, which are considered stem markers, compared to the parental adherent cells (S6 Fig)

**Fig 9:**
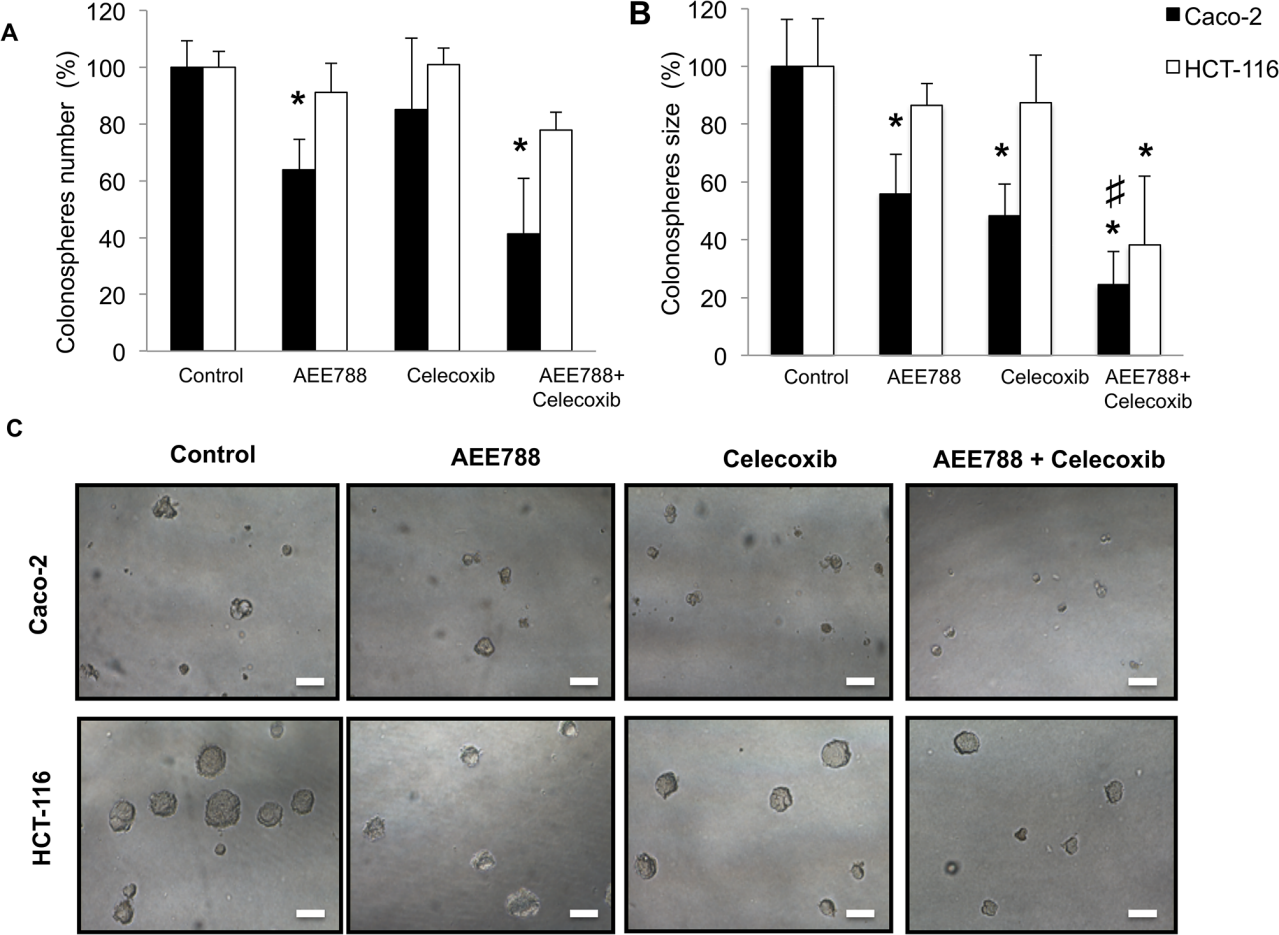
Combined AEE788/ Celecoxib treatment in colon cancer cells impairs colonosphere formation capability. Colon cancer cells were pre-treated with 2.5 µM AEE788 as a single agent or in combination with 10 µM celecoxib in the presence of 100 ng/mL EGF for 48h, and then cells were seeded at clonal density with serum free medium in low-adherence plates. After seven days, the number (A), size (B) and appearance (C) of formed colonospheres were evaluated by light microscopy. Spheres size was quantified in micrograph with the imaging software (Image J software). (Final magnification: X100, scale bar corresponds to 100 microns. Data are means ± SEM of three independent experiments (*p <0.05, compared with the control; # p<0.05, compared with AEE788-treated cells).

As shown in Fig 9A, pretreatment of Caco-2 cells with AEE788, alone or in combination with celecoxib, reduced their capability to form colonospheres. On the contrary, only combined treatment significantly reduced the formation of colonospheres in the case of HCT-116 cells. Therefore, in both cell lines the combined treatment contributed to the diminution of the CSC subpopulation.

**Fig 10:**
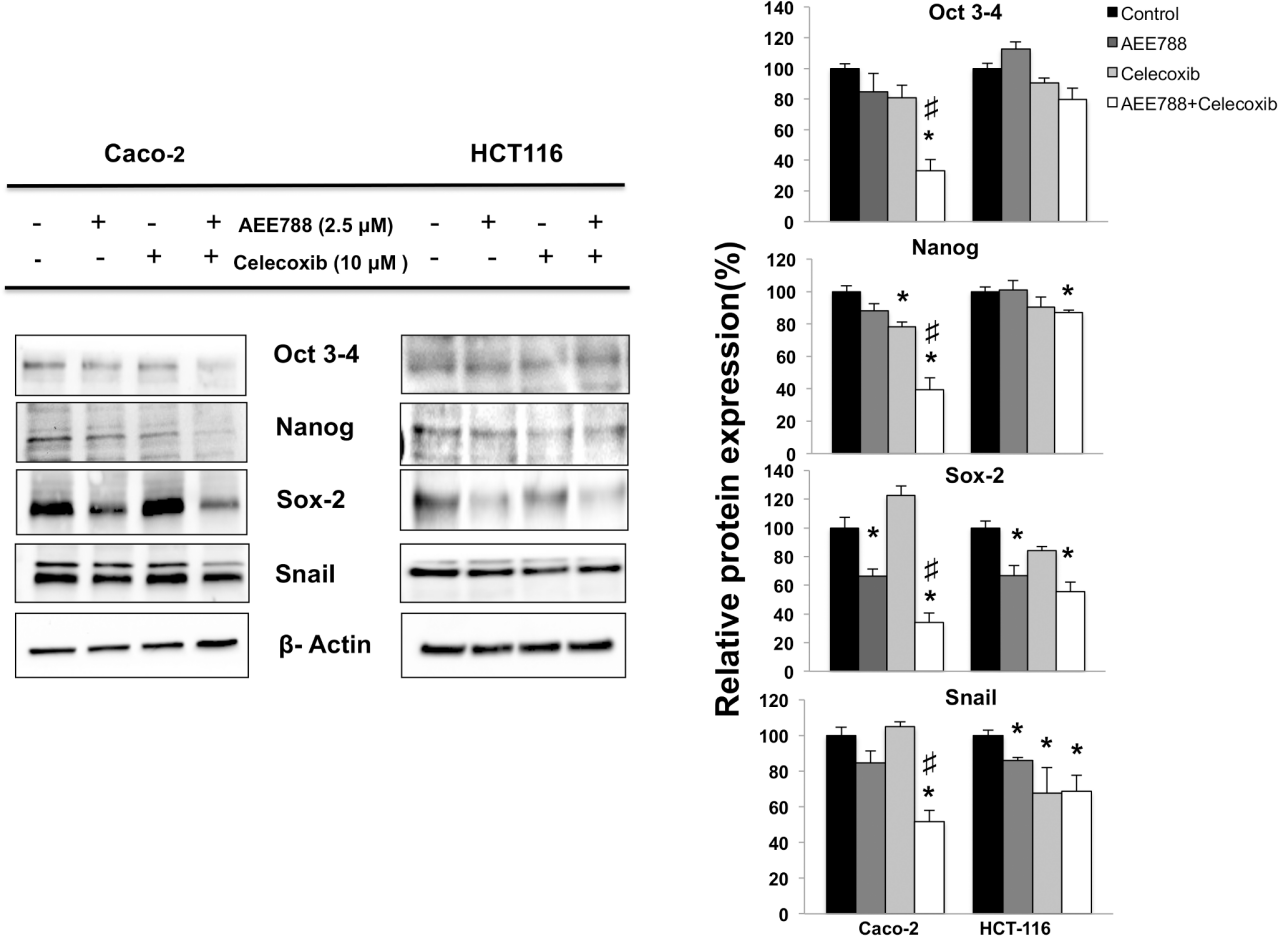
Combined AEE788/celecoxib treatment downregulates stemness-related pathways in colorectal cancer cells. The expression of stem cell markers Oct 3/4, Nanog and Sox-2 was analyzed by western blot in total cell extracts of colon cancer cells after 6h of indicated treatments. The expression of -actin is included as loading control. The corresponding densitometric analysis is also shown. Data are means ± SEM of three independent experiments (*p <0.05, compared with the control; # p<0.05, compared with AEE788-treated cells).

Besides, in both cell lines the combined treatment caused the formation of lower size colonospheres compared to those derived from untreated cells (Fig 9B and 9C). This notion is in agreement with data of expression levels of stem cell markers Oct 3/4, Nanog, Sox-2 and Snail, which were significantly reduced in Caco-2 cells after treatment with AEE788, and specially when combined with celecoxib (Fig 10). Reduced expression of Nanog and Sox-2 was also found in HCT116 cells after treatment with AEE788 alone or in combination with celecoxib. Taken together, these data suggest that these treatments are effective in the reduction of colon CSCs subpopulation in both K-Ras mutated and K-Ras wildtype cells by targeting stemness-related pathways.

## Discussion

Numerous complex biochemical pathways participate in tumor cell proliferation and angiogenesis, and combination therapies designed to target multiple oncogenic pathways are likely to be more effective. In our study, the combination of AEE788, which is a dual receptor TKI of both EGFR and VEGFR, with the specific COX-2 inhibitor celecoxib not only demonstrated enhanced efficacy to inhibit colon cancer cell proliferation, migration and angiogenesis but also reduced colon CSCs subpopulation by targeting stemness-related pathways.

AEE788 has been reported previously to exert both apoptotic effects and cell cycle arrest in various human cancer models [16–18]. Consistent with these data, we here show that AEE788 impaired EGFR downstream signaling, inhibiting cell proliferation, augmenting apoptotic cell death and causing cell cycle arrest at G1 phase in Caco-2 cells, but not in K-Ras mutated HCT116 cells. Accordingly, the transfection of mutant K-Ras into Caco-2 cells confirmed that downstream activation of the EGFR-Ras-ERK pathway, abolished the antiproliferative activity of AEE788. Therefore, the anti-tumoral activity of AEE788, alone or in combination with celecoxib, in colon cancer cells was strongly dependent on wild-type K-Ras status.

Several studies have shown that tumour cells express VEGF receptors and respond to autocrine and paracrine VEGF signals [19]. Our results support this hypothesis, since AEE788 was able to inhibit VEGF-driven proliferation in Caco-2 cells. However, AEE788 is a weaker VEGFR inhibitor as compared with its anti-EGFR activity [15]. In this regard, a pharmacodynamic-based study showed effective inhibition of EGFR, but not of VEGFR at tolerable AEE788 doses, which led to the termination of the AEE788 drug development program [30]. On the other hand, an autocrine loop involving VEGF induction of COX-2 expression and prostaglandin synthesis, which in turn induces VEGF production has been described [20,21]. Notably, we have shown that the combination with COX-2 inhibitors intensified the antitumoral effects of AEE788. Moreover, the existence of this autocrine VEGF signalling is supported by our observation that the inhibition of VEGF-A mRNA levels, VEGF165 protein secretion, and angiogenic activity of conditioned medium in AEE788-treated Caco-2 cells was significantly intensified by celecoxib. COX-2 is a pleiotropic enzyme that mediates many cellular functions, including cell motility [31,32], and accordingly our data shows that celecoxib efficiently inhibited Caco-2 cells migration. Moreover, the combined treatment of celecoxib with AEE788 had a greater impact on cancer cell migration in agreement with previous studies suggesting crosstalk between EGFR and COX-2 [33].

It is known that prostaglandin E2, a COX-2-derived eicosanoid, promotes colon cancer cell growth by strengthening wnt/β-catenin signaling [34]. Besides, the biological effects of PGE2 are mediated by EP receptors, which has been reported to transactivate EGFR [35,36]. Therefore, the crosstalk between EGFR and COX-2 may also have an important role in regulating stemness-related pathways, such as wnt/β-catenin pathway, in colon cancer. In supporting this notion, we found that the combined AEE788/celecoxib treatment drastically reduced nuclear β-catenin levels in colon cancer cells. Although the molecular mechanisms underlying the β-catenin nuclear localization remain unclear, a recent study have shown that the direct interaction of forkhead box M1 (FOXM1) transcription factor with β-catenin is necessary and sufficient for its nuclear localization and transcriptional activation in glioma tumor cells [26]. Interestingly, EGFR/RAS signaling leads to FOXM1 activation and nuclear translocation [37,38], and our data support that the capability of the combined inhibition of EGFR and COX-2 to impair the nuclear localization of β-catenin may be related to the disturbance of this FOXM1/β-catenin axis. Indeed, our study demonstrates that the combined AEE788/celecoxib treatment reduces the interaction of both proteins, as indicated by the co-immunoprecipitation assays. This will also explain why AEE788 was more effective in the reduction of stem characteristic in colon cancer cells.

The presence of cancer stem cells (CSCs) in tumours is likely one of the main reasons why current oncologic therapies are poorly effective in preventing tumour progression, metastasis and recurrence [10]. Therefore selective elimination of CSCs may become a necessary step for an effective treatment in colorectal cancer [39]. In our study, the combined treatment of AEE788 with celecoxib significantly reduced the capacity of colon cancer cells to grow forming colonospheres, a functional assay for the ability of self-renewal that is characteristic of CSCs. This effect may be related to the impact that this treatment had on β-catenin subcellular location. Wnt/β-catenin signaling plays a critical role in CSCs, including colon CSCs [39], and previous studies have shown that levels of β-catenin regulate the activity of pluripotent markers such as Oct3/4, Nanog and Sox2 [40–42]. Accordingly, we here have shown that the treatment with AEE788, specially when combined with celecoxib, significantly reduced the expression of Oct 3/4, Nanog and Sox-2 in colon cancer cells. Importantly, the increased expression of either SOX2 or nuclear β-catenin have been shown to be associated with distant metastases in colorectal cancer [43], underlining the importance of these stemness-associated factors for distant spread in this disease.

In summary, our study shows that the combined treatment of AEE788 and celecoxib not only demonstrated enhanced anti-tumoral efficacy in colorectal cancer cells, but also reduced colon CSCs subpopulation by targeting stemness-related pathways. Our data support the notion that the combined inhibition of EGFR and COX-2 in colorectal cancer cells disturbs FOXM1/β-catenin axis impacting CSC subpopulation, and therefore might improve the efficacy of existing therapies in colorectal cancer.

## Supporting Information

S1 Fig AEE788 Inhibits VEGF-driven cell proliferation in colorectal cancer cells. VEGF-driven (50 ng/ml) cell proliferation was evaluated after 48 h of treatment with different doses of AEE788. Data are means ± SEM of three independent experiments (*p <0.05, compared with the control).
(TIF)

S2 Fig Transient expression of mutant K-RAS gene in Caco-2 cells reduces the antiproliferative effect of AEE788. Caco-2 cells were transiently transfected with pBabe K-Ras 12V plasmid and the EGF-independent ERK1/2 phosphorylation confirmed downstream activation of the EGFR-Ras-ERK pathway in these cells **(A).** Cells transfected with mutant (12V) K-Ras showed reduced sensitivity to the antiproliferative effect of AEE788 **(B).** Data are means ± SEM of three independent experiments (*p <0.05).
(TIF)

S3 Fig Prostaglandin E2 (PGE2) production and cyclooxygenase-2 (COX-2) expression in AEE788/celecoxib-treated colorectal cancer cells. PGE2 levels were evaluated in whole cell lysates after 12 h of the indicated treatments. Data are means ± SEM of three independent experiments (*p <0.05, compared with the control) **(A).** Cells were treated for 6 h to the indicated treatments and COX-2 expression was analyzed by western-blot in whole cell extracts. Expression of β-actin is included as loading control.
(TIF)

S4 Fig The phosphorylated and non-phosphorylated forms of EGFR, VEGFR2, ERK 1/2, AKT and Stat3 were detected using an antibody array kit (as described under Material and Methods) in cells grown in the presence of EGF (100 ng/mL) and treated with AEE788 (2.5 µM) and/or celecoxib (10 μM) for 6h. The array images were captured and quantification of phosphorylated forms ((normalized to their corresponding non-phosphorylated counterparts) was done using Image-Lab software (Biorad-Molecular Images, ChemiDoc XRS). Data are means ± SEM of three independent experiments (*p <0.05, compared with the control).
(TIF)

S5 Fig Formed colonospheres are derived from single cells. Lipophilic fluorescent labeling was performed to confirm that individual colonospheres were derived from single cells. Equal numbers of DiI (Red)- or DiO (Green)-labelled cells were mixed prior to seeding at clonal density to perform the colonosphere formation assay, as described under Materials and Methods. The assay resulted in the formation of DiI (Red)- or DiO (Green)-labelled spheres, whereas mixed labeled colonospheres were not observed, thus confirming that tumorospheres are derived from single cells. (Final magnification: X200, scale bar corresponds to 100 microns).
(TIF)

S6 Fig Colonospheres formed by Caco-2 and HCT-116 cells have increased expression of pluripotency-related proteins. **A)** The expression of the stem-related proteins Oct 3/4, Nanog and SOX-2 were analyzed in total cell extracts using an antibody array as described in Materials and Methods. Data are shown as fold change in cells growing as colonospheres compared to parental adherent cell cultures. **B)** The expression of β-Catenin and Ep-CAM was analyzed in both Caco-2 and HCT-116 cells grown as colonospheres and parental adherent growing cells spheres. The expression of -actin is included as loading control. Data are means ± SEM of three independent experiments (*p <0.05, compared with the control).
(TIF)

## References

[1] Ferlay J, Shin HR, Bray F, Forman D, Mathers C, Parkin DM. Estimates of worldwide burden of cancer in 2008: GLOBOCAN 2008. Int J Cancer 2010 12 15;127(12):2893–2917. 10.1002/ijc.25516 21351269

[2] Ferlay J, Steliarova-Foucher E, Lortet-Tieulent J, Rosso S, Coebergh JW, Comber H, et al Cancer incidence and mortality patterns in Europe: estimates for 40 countries in 2012. Eur J Cancer 2013 4;49(6):1374–1403. 10.1016/j.ejca.2012.12.027 23485231

[3] Siena S, Sartore-Bianchi A, Di NF, Balfour J, Bardelli A. Biomarkers predicting clinical outcome of epidermal growth factor receptor-targeted therapy in metastatic colorectal cancer. J Natl Cancer Inst 2009 10 7;101(19):1308–1324. 10.1093/jnci/djp280 19738166 PMC2758310

[4] Bardelli A, Siena S. Molecular mechanisms of resistance to cetuximab and panitumumab in colorectal cancer. J Clin Oncol 2010 3 1;28(7):1254–1261. 10.1200/JCO.2009.24.6116 20100961

[5] Hubbard J, Grothey A. Antiangiogenesis agents in colorectal cancer. Curr Opin Oncol 2010 7;22(4):374–380. 10.1097/CCO.0b013e328339524e 20375894

[6] Marques I, Araujo A, de Mello RA. Anti-angiogenic therapies for metastatic colorectal cancer: current and future perspectives. World J Gastroenterol 2013 11 28;19(44):7955–7971. 10.3748/wjg.v19.i44.7955 24307789 PMC3848143

[7] Mulder K, Scarfe A, Chua N, Spratlin J. The role of bevacizumab in colorectal cancer: understanding its benefits and limitations. Expert Opin Biol Ther 2011 3;11(3):405–413. 10.1517/14712598.2011.557657 21281258

[8] Vermeulen L, De Sousa E Melo, Richel DJ, Medema JP. The developing cancer stem-cell model: clinical challenges and opportunities. Lancet Oncol 2012 2;13(2):e83–e89. 10.1016/S1470-2045(11)70257-1 22300863

[9] Visvader JE, Lindeman GJ. Cancer stem cells: current status and evolving complexities. Cell Stem Cell 2012 6 14;10(6):717–728. 10.1016/j.stem.2012.05.007 22704512

[10] Merlos-Suarez A, Barriga FM, Jung P, Iglesias M, Cespedes MV, Rossell D, et al The intestinal stem cell signature identifies colorectal cancer stem cells and predicts disease relapse. Cell Stem Cell 2011 5 6;8(5):511–524. 10.1016/j.stem.2011.02.020 21419747

[11] Luraghi P, Reato G, Cipriano E, Sassi F, Orzan F, Bigatto V, et al MET signaling in colon cancer stem-like cells blunts the therapeutic response to EGFR inhibitors. Cancer Res 2014 3 15;74(6):1857–1869. 10.1158/0008-5472.CAN-13-2340-T 24448239

[12] Zhao D, Pan C, Sun J, Gilbert C, Drews-Elger K, Azzam DJ, et al. VEGF drives cancer-initiating stem cells through VEGFR-2/Stat3 signaling to upregulate Myc and Sox2. Oncogene 2014 Aug 25. 10.1038/onc.2014.257 25151964

[13] Moreira L, Castells A. Cyclooxygenase as a target for colorectal cancer chemoprevention. Curr Drug Targets 2011 12;12(13):1888–1894. 21158711 10.2174/138945011798184218

[14] Xu L, Stevens J, Hilton MB, Seaman S, Conrads TP, Veenstra TD, et al COX-2 inhibition potentiates antiangiogenic cancer therapy and prevents metastasis in preclinical models. Sci Transl Med 2014 6 25;6(242):242ra84. 10.1126/scitranslmed.3008455 PMC6309995 24964992

[15] Traxler P, Allegrini PR, Brandt R, Brueggen J, Cozens R, Fabbro D, et al AEE788: a dual family epidermal growth factor receptor/ErbB2 and vascular endothelial growth factor receptor tyrosine kinase inhibitor with antitumor and antiangiogenic activity. Cancer Res 2004 7 15;64(14):4931–4941. 15256466 10.1158/0008-5472.CAN-03-3681

[16] Park YW, Younes MN, Jasser SA, Yigitbasi OG, Zhou G, Bucana CD, et al AEE788, a dual tyrosine kinase receptor inhibitor, induces endothelial cell apoptosis in human cutaneous squamous cell carcinoma xenografts in nude mice. Clin Cancer Res 2005 3 1;11(5):1963–1973. 15756022 10.1158/1078-0432.CCR-04-1665

[17] Barbarroja N, Torres LA, Rodriguez-Ariza A, Valverde-Estepa A, Lopez-Sanchez LM, Ruiz-Limon P, et al AEE788 is a vascular endothelial growth factor receptor tyrosine kinase inhibitor with antiproliferative and proapoptotic effects in acute myeloid leukemia. Exp Hematol 2010 8;38(8):641–652. 10.1016/j.exphem.2010.03.017 20380868

[18] Venkatesan P, Bhutia SK, Singh AK, Das SK, Dash R, Chaudhury K, et al AEE788 potentiates celecoxib-induced growth inhibition and apoptosis in human colon cancer cells. Life Sci 2012 10 22;91(15–16):789–799. 10.1016/j.lfs.2012.08.024 22922496

[19] Goel HL, Mercurio AM. VEGF targets the tumour cell. Nat Rev Cancer 2013 12;13(12):871–882. 10.1038/nrc3627 24263190 PMC4011842

[20] Grau R, Punzon C, Fresno M, Iniguez MA. Peroxisome-proliferator-activated receptor alpha agonists inhibit cyclo-oxygenase 2 and vascular endothelial growth factor transcriptional activation in human colorectal carcinoma cells via inhibition of activator protein-1. Biochem J 2006 4 1;395(1):81–88. 16343055 10.1042/BJ20050964 PMC1409694

[21] Toomey DP, Murphy JF, Conlon KC. COX-2, VEGF and tumour angiogenesis. Surgeon 2009 6;7(3):174–180. 19580182 10.1016/s1479-666x(09)80042-5

[22] Oommen S, Gupta SK, Vlahakis NE. Vascular endothelial growth factor A (VEGF-A) induces endothelial and cancer cell migration through direct binding to integrin {alpha}9{beta}1: identification of a specific {alpha}9{beta}1 binding site. J Biol Chem 2011 1 14;286(2):1083–1092. 10.1074/jbc.M110.175158 21071450 PMC3020715

[23] Perrot-Applanat M, Di BM. Autocrine functions of VEGF in breast tumor cells: adhesion, survival, migration and invasion. Cell Adh Migr 2012 11;6(6):547–553. 10.4161/cam.23332 23257828 PMC3547902

[24] Anastas JN, Moon RT. WNT signalling pathways as therapeutic targets in cancer. Nat Rev Cancer 2013 1;13(1):11–26. 10.1038/nrc3419 23258168

[25] Henderson BR, Fagotto F. The ins and outs of APC and beta-catenin nuclear transport. EMBO Rep 2002 9;3(9):834–839. 12223464 10.1093/embo-reports/kvf181 PMC1084234

[26] Zhang N, Wei P, Gong A, Chiu WT, Lee HT, Colman H, et al FoxM1 promotes beta-catenin nuclear localization and controls Wnt target-gene expression and glioma tumorigenesis. Cancer Cell 2011 10 18;20(4):427–442. 10.1016/j.ccr.2011.08.016 22014570 PMC3199318

[27] Fodde R, Brabletz T. Wnt/beta-catenin signaling in cancer stemness and malignant behavior. Curr Opin Cell Biol 2007 4;19(2):150–158. 17306971 10.1016/j.ceb.2007.02.007

[28] Vermeulen L, De Sousa E Melo, van der HM, Cameron K, de Jong JH, Borovski T, et al Wnt activity defines colon cancer stem cells and is regulated by the microenvironment. Nat Cell Biol 2010 5;12(5):468–476. 10.1038/ncb2048 20418870

[29] Holland JD, Klaus A, Garratt AN, Birchmeier W. Wnt signaling in stem and cancer stem cells. Curr Opin Cell Biol 2013 4;25(2):254–264. 10.1016/j.ceb.2013.01.004 23347562

[30] Baselga J, Mita AC, Schoffski P, Dumez H, Rojo F, Tabernero J, et al Using pharmacokinetic and pharmacodynamic data in early decision making regarding drug development: a phase I clinical trial evaluating tyrosine kinase inhibitor, AEE788. Clin Cancer Res 2012 11 15;18(22):6364–6372. 10.1158/1078-0432.CCR-12-1499 23014528

[31] Banu N, Buda A, Chell S, Elder D, Moorghen M, Paraskeva C, et al Inhibition of COX-2 with NS-398 decreases colon cancer cell motility through blocking epidermal growth factor receptor transactivation: possibilities for combination therapy. Cell Prolif 2007 10;40(5):768–779. 17877615 10.1111/j.1365-2184.2007.00459.x PMC6496834

[32] Larkins TL, Nowell M, Singh S, Sanford GL. Inhibition of cyclooxygenase-2 decreases breast cancer cell motility, invasion and matrix metalloproteinase expression. BMC Cancer 2006;6:181 16831226 10.1186/1471-2407-6-181 PMC1559713

[33] Dannenberg AJ, Lippman SM, Mann JR, Subbaramaiah K, DuBois RN. Cyclooxygenase-2 and epidermal growth factor receptor: pharmacologic targets for chemoprevention. J Clin Oncol 2005 1 10;23(2):254–266. 15637389 10.1200/JCO.2005.09.112

[34] Castellone MD, Teramoto H, Williams BO, Druey KM, Gutkind JS. Prostaglandin E2 promotes colon cancer cell growth through a Gs-axin-beta-catenin signaling axis. Science 2005 12 2;310(5753):1504–1510. 16293724 10.1126/science.1116221

[35] Han C, Michalopoulos GK, Wu T. Prostaglandin E2 receptor EP1 transactivates EGFR/MET receptor tyrosine kinases and enhances invasiveness in human hepatocellular carcinoma cells. J Cell Physiol 2006 4;207(1):261–270. 16331686 10.1002/jcp.20560

[36] Donnini S, Finetti F, Solito R, Terzuoli E, Sacchetti A, Morbidelli L, et al EP2 prostanoid receptor promotes squamous cell carcinoma growth through epidermal growth factor receptor transactivation and iNOS and ERK1/2 pathways. FASEB J 2007 8;21(10):2418–2430. 17384145 10.1096/fj.06-7581com

[37] Ma RY, Tong TH, Leung WY, Yao KM. Raf/MEK/MAPK signaling stimulates the nuclear translocation and transactivating activity of FOXM1. Methods Mol Biol 2010;647:113–123. 10.1007/978-1-60761-738-9_6 20694663

[38] Wang IC, Ustiyan V, Zhang Y, Cai Y, Kalin TV, Kalinichenko VV. Foxm1 transcription factor is required for the initiation of lung tumorigenesis by oncogenic Kras. Oncogene 2014 11 13;33(46):5391–5396. 10.1038/onc.2013.475 24213573

[39] Botchkina G. Colon cancer stem cells—from basic to clinical application. Cancer Lett 2013 9 10;338(1):127–140. 10.1016/j.canlet.2012.04.006 22537805

[40] Wang Y, Dong J, Li D, Lai L, Siwko S, Li Y, et al Lgr4 regulates mammary gland development and stem cell activity through the pluripotency transcription factor Sox2. Stem Cells 2013 9;31(9):1921–1931. 10.1002/stem.1438 23712846 PMC3934111

[41] Faunes F, Hayward P, Descalzo SM, Chatterjee SS, Balayo T, Trott J, et al A membrane-associated beta-catenin/Oct4 complex correlates with ground-state pluripotency in mouse embryonic stem cells. Development 2013 3;140(6):1171–1183. 10.1242/dev.085654 23444350 PMC3585656

[42] Li XQ, Yang XL, Zhang G, Wu SP, Deng XB, Xiao SJ, et al Nuclear beta-catenin accumulation is associated with increased expression of Nanog protein and predicts poor prognosis of non-small cell lung cancer. J Transl Med 2013;11:114 10.1186/1479-5876-11-114 23648139 PMC3706347

[43] Neumann J, Bahr F, Horst D, Kriegl L, Engel J, Luque RM, et al SOX2 expression correlates with lymph-node metastases and distant spread in right-sided colon cancer. BMC Cancer 2011;11:518 10.1186/1471-2407-11-518 22168803 PMC3267776

