# Supplementary figures and images for: Simultaneous Inhibition of EGFR/VEGFR and Cyclooxygenase-2 Targets Stemness-Related Pathways in Colorectal Cancer Cells
Source: PLoS One. 2015 Jun 24;10(6):e0131363. doi: 10.1371/journal.pone.0131363 (PMC4479446; doi:10.1371/journal.pone.0131363)

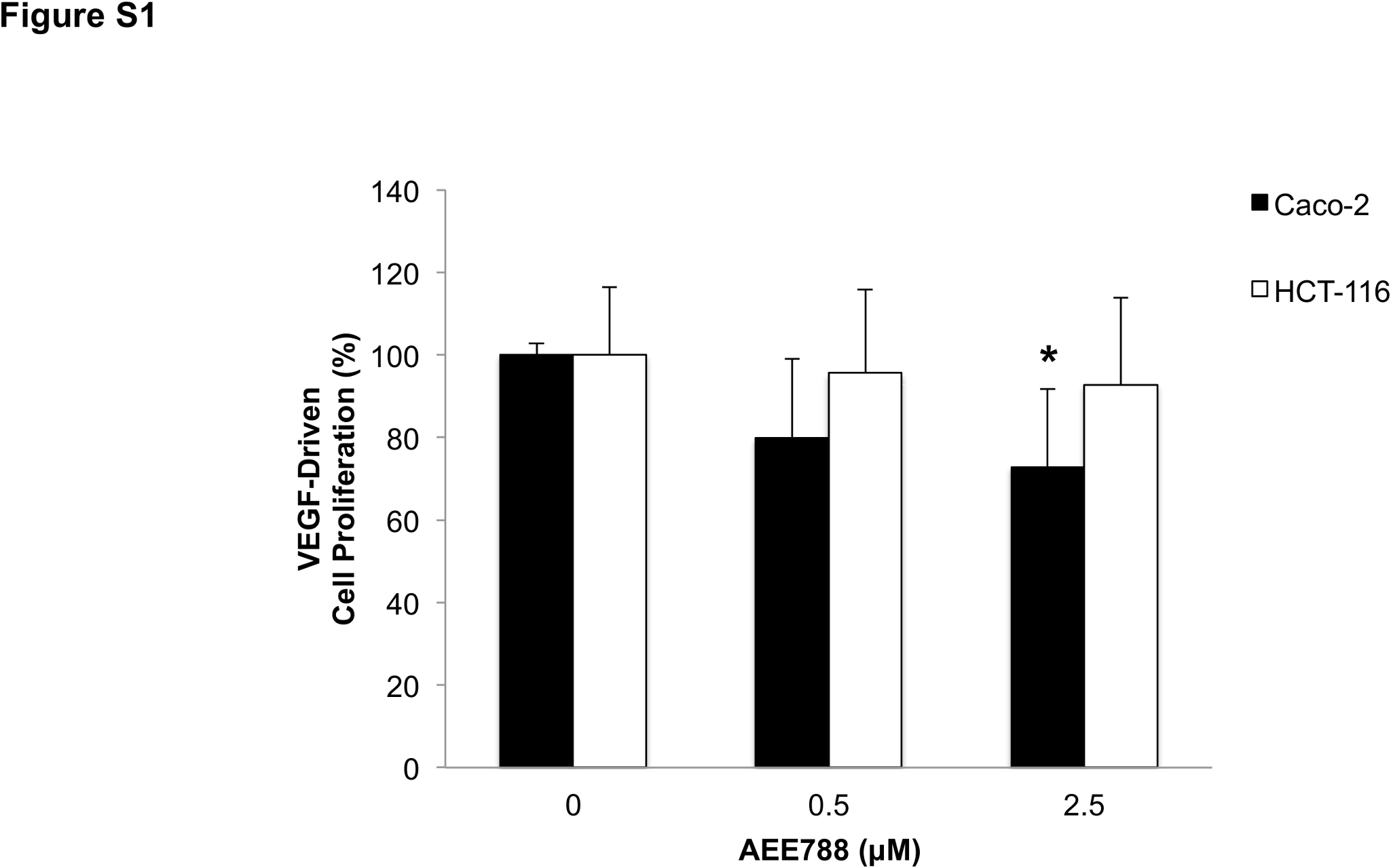

Supplement: S1 Fig — VEGF-driven (50 ng/ml) cell proliferation was evaluated after 48 h of treatment with different doses of AEE788. Data are means ± SEM of three independent experiments (*p <0.05, compared with the control). (TIF) [file pone.0131363.s001.tif]

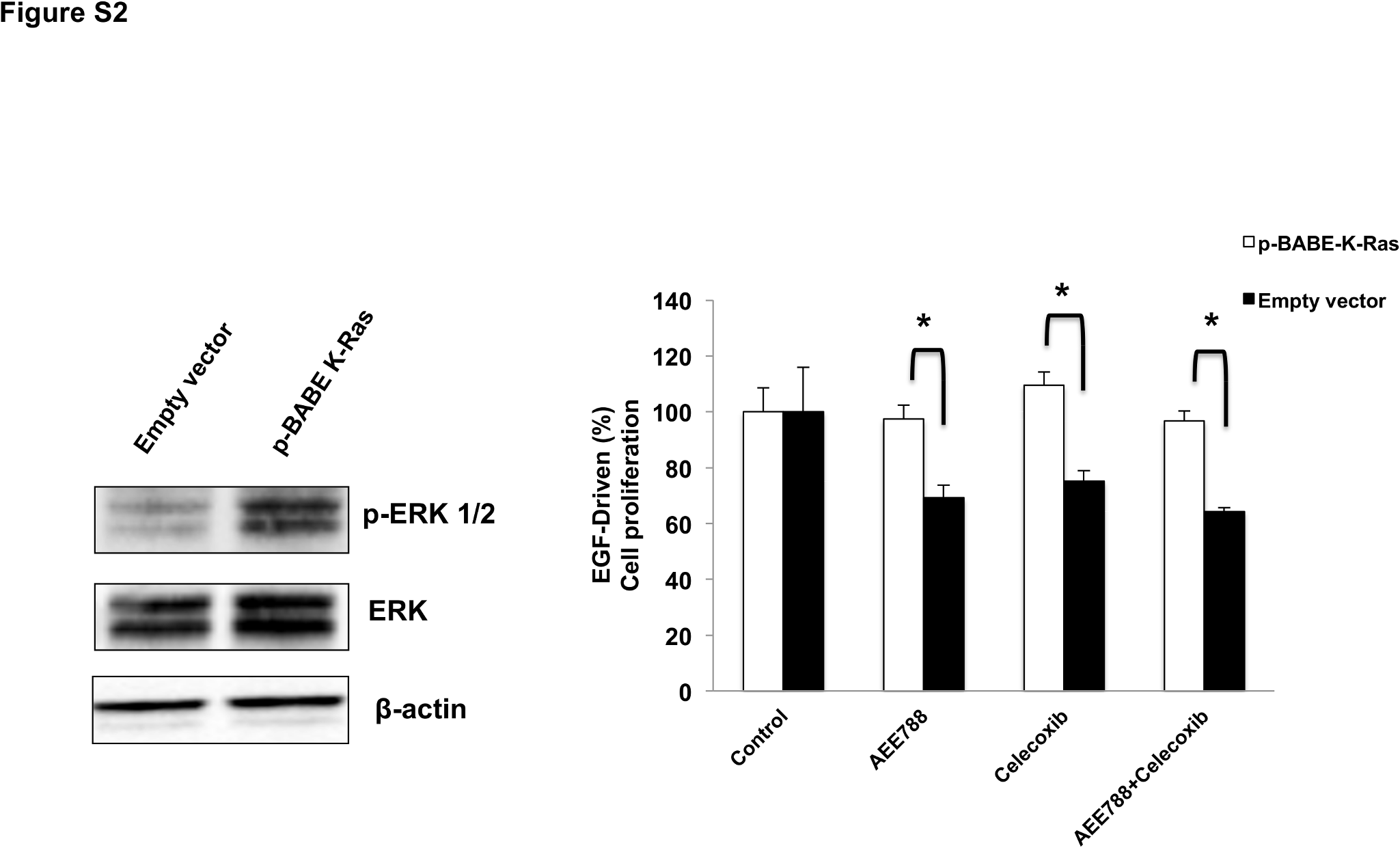

Supplement: S2 Fig — Caco-2 cells were transiently transfected with pBabe K-Ras 12V plasmid and the EGF-independent ERK1/2 phosphorylation confirmed downstream activation of the EGFR-Ras-ERK pathway in these cells (A). Cells transfected with mutant (12V) K-Ras showed reduced sensitivity to the antiproliferative effect of AEE788 (B). Data are means ± SEM of three independent experiments (*p <0.05). (TIF) [file pone.0131363.s002.tif]

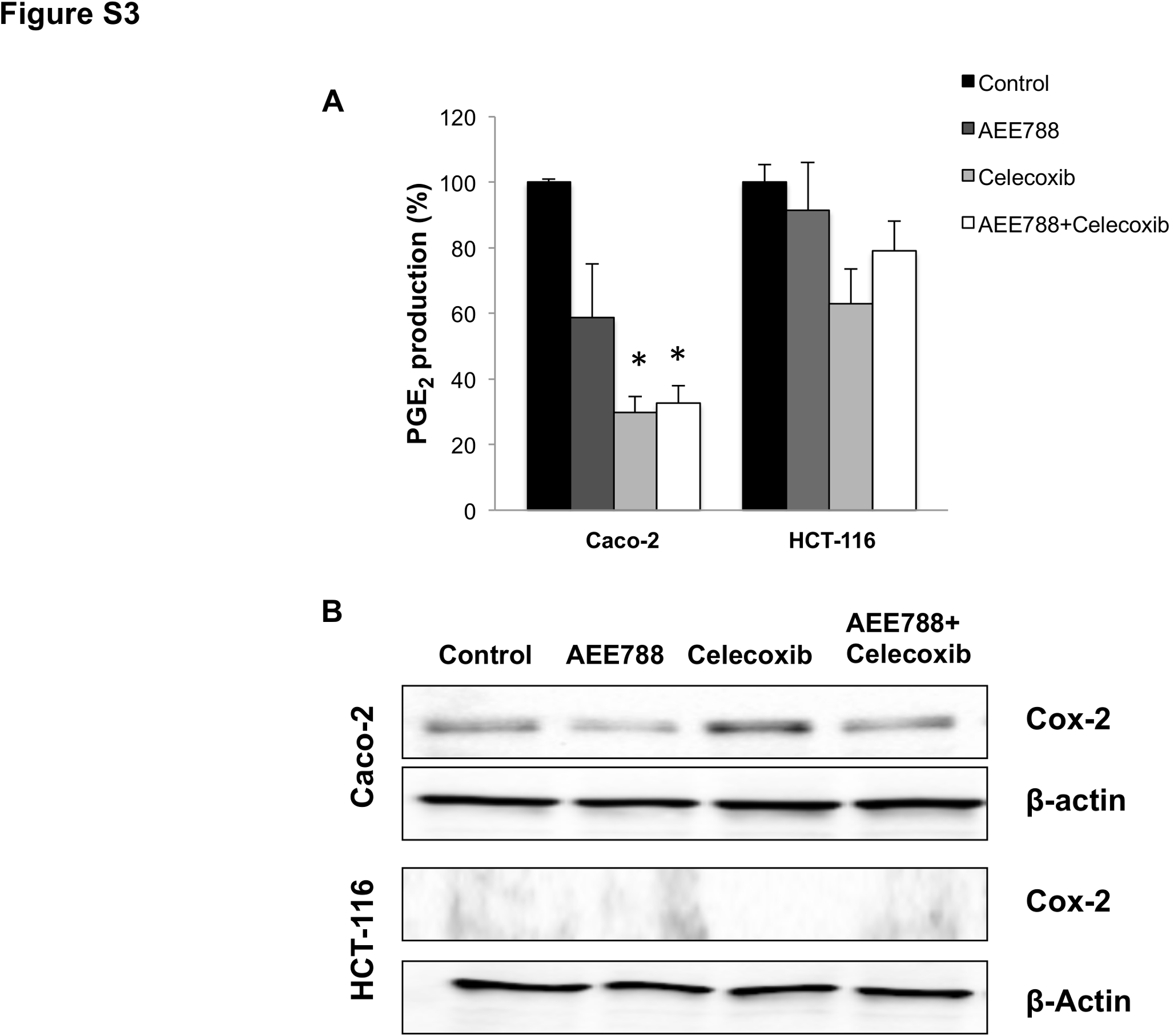

Supplement: S3 Fig — PGE2 levels were evaluated in whole cell lysates after 12 h of the indicated treatments. Data are means ± SEM of three independent experiments (*p <0.05, compared with the control) (A). Cells were treated for 6 h to the indicated treatments and COX-2 expression was analyzed by western-blot in whole cell extracts. Expression of β-actin is included as loading control. (TIF) [file pone.0131363.s003.tif]

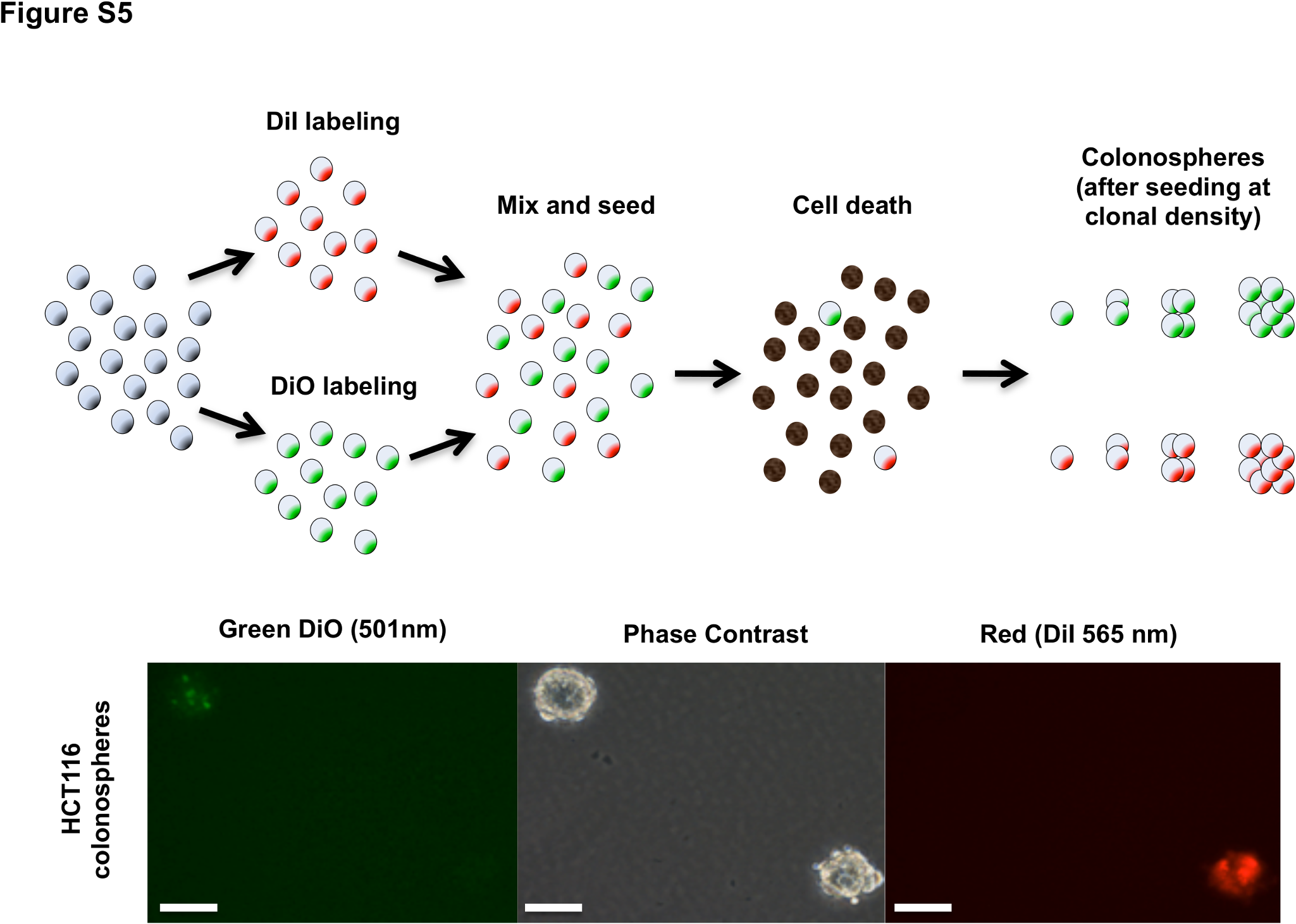

Supplement: S5 Fig — Lipophilic fluorescent labeling was performed to confirm that individual colonospheres were derived from single cells. Equal numbers of DiI (Red)- or DiO (Green)-labelled cells were mixed prior to seeding at clonal density to perform the colonosphere formation assay, as described under Materials and Methods. The assay resulted in the formation of DiI (Red)- or DiO (Green)-labelled spheres, whereas mixed labeled colonospheres were not observed, thus confirming that tumorospheres are derived from single cells. (Final magnification: X200, scale bar corresponds to 100 microns). (TIF) [file pone.0131363.s005.tif]

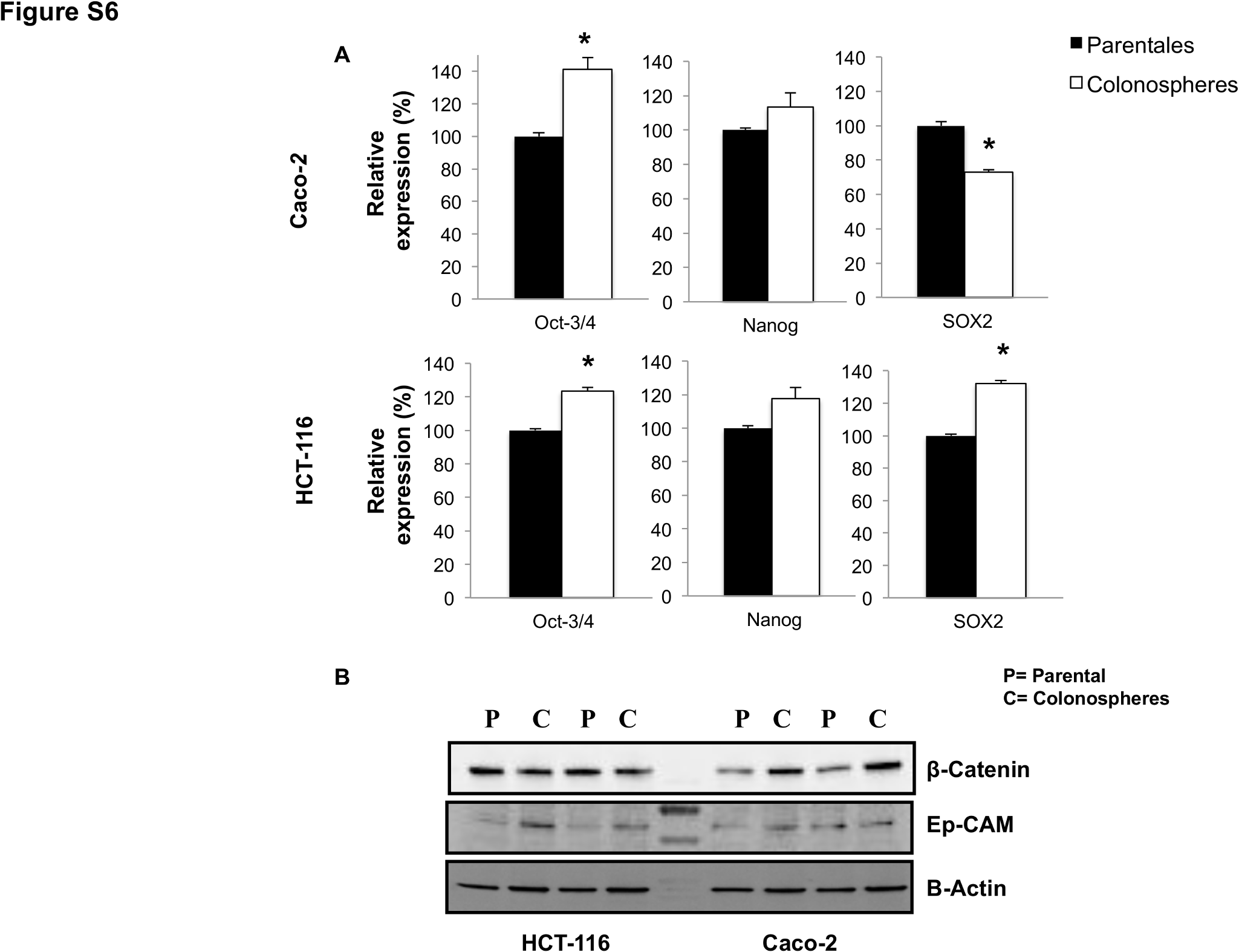

Supplement: S6 Fig — A) The expression of the stem-related proteins Oct 3/4, Nanog and SOX-2 were analyzed in total cell extracts using an antibody array as described in Materials and Methods. Data are shown as fold change in cells growing as colonospheres compared to parental adherent cell cultures. B) The expression of β-Catenin and Ep-CAM was analyzed in both Caco-2 and HCT-116 cells grown as colonospheres and parental adherent growing cells spheres. The expression of -actin is included as loading control. Data are means ± SEM of three independent experiments (*p <0.05, compared with the control). (TIF) [file pone.0131363.s006.tif]
